# Transparent reporting of multivariable prediction models developed or validated using clustered data (TRIPOD-Cluster): explanation and elaboration

**DOI:** 10.1136/bmj-2022-071058

**Published:** 2023-02-07

**Authors:** Thomas P A Debray, Gary S Collins, Richard D Riley, Kym I E Snell, Ben Van Calster, Johannes B Reitsma, Karel G M Moons

**Affiliations:** 1 Julius Center for Health Sciences and Primary Care, University Medical Center Utrecht, Utrecht University, Utrecht, Netherlands; 2 Cochrane Netherlands, University Medical Center Utrecht, Utrecht University, Utrecht, Netherlands; 3 Centre for Statistics in Medicine, Nuffield Department of Orthopaedics, Rheumatology and Musculoskeletal Sciences, Botnar Research Centre, University of Oxford, Oxford, UK; 4 National Institute for Health and Care Research Oxford Biomedical Research Centre, John Radcliffe Hospital, Oxford, UK; 5 Centre for Prognosis Research, School of Medicine, Keele University, Keele, UK; 6 Department of Development and Regeneration, KU Leuven, Leuven, Belgium; 7 EPI-centre, KU Leuven, Leuven, Belgium; 8 Department of Biomedical Data Sciences, Leiden University Medical Center, Leiden, Netherlands

## Abstract

The TRIPOD-Cluster (transparent reporting of multivariable prediction models developed or validated using clustered data) statement comprises a 19 item checklist, which aims to improve the reporting of studies developing or validating a prediction model in clustered data, such as individual participant data meta-analyses (clustering by study) and electronic health records (clustering by practice or hospital). This explanation and elaboration document describes the rationale; clarifies the meaning of each item; and discusses why transparent reporting is important, with a view to assessing risk of bias and clinical usefulness of the prediction model. Each checklist item of the TRIPOD-Cluster statement is explained in detail and accompanied by published examples of good reporting. The document also serves as a reference of factors to consider when designing, conducting, and analysing prediction model development or validation studies in clustered data. To aid the editorial process and help peer reviewers and, ultimately, readers and systematic reviewers of prediction model studies, authors are recommended to include a completed checklist in their submission.

Medical decisions are often guided by predicted probabilities, for example, regarding the presence of a specific disease or condition (diagnosis) or that a specific outcome will occur in time (prognosis).^1^ ^2^ ^3^ ^4^ ^5^ Predicted probabilities are typically estimated using multivariable models by combining information or values from multiple variables (called predictors) that are observed or measured in an individual. Prediction models are typically aimed at assisting healthcare professions in making clinical decisions for individual patients.^5^ ^6^ In essence, a prediction model is an equation that converts an individual’s observed predictor values into a probability (or risk) of a particular outcome occurring.

Prediction models fall into two broad categories: diagnostic and prognostic (box 1; fig 1).^1^ ^3^ In a diagnostic model, two or more predictors are combined and used to estimate the probability that a certain condition is present at the moment of prediction: a cross sectional relation or prediction. They are typically developed for individuals suspected of having that condition based on presenting symptoms or signs. In a prognostic model, multiple predictors are combined and used to estimate the probability of a particular event occurring within a given prediction (time) horizon: a longitudinal relation or prediction. Prognostic models are developed for individuals in a particular health state that are at risk of developing the outcome of interest.^5^ ^8^ ^9^ We use the term “prognostic models” in the broad sense, referring to the prediction of a future condition in individuals with symptoms or in those without (eg, individuals in the general population), rather than the narrower definition of predicting the disease course of patients receiving a diagnosis of a particular disease with or without treatment. The prediction horizon can vary considerably depending on the event of interest. For example, when predicting in-hospital complications after surgery, the horizon is shorter than for predicting mortality at three months in patients receiving a diagnosis of pancreatic cancer, which in turn is shorter than predicting outcomes such as coronary heart disease in the general population where the prediction horizon is often 10 years.^10^

Box 1 Types of prediction model studies, adapted from TRIPOD E&E 2015^2^*

**Prediction model development studies**

These studies aim to develop or produce a prediction model by identifying predictors of the outcome (eg, based on a priori knowledge, data driven analysis) that can be used for tailored predictions by estimating the weight (or coefficient) of each predictor. Sometimes, the development can focus on updating an existing prediction model by including one or more additional predictors, for example, that were identified following the development of the original model.

Quantification of the model’s predictive performance (eg, by calibration, discrimination, clinical utility) using the same dataset in which the model was developed (often referred to as apparent performance) will tend to give optimistic results, particularly in small datasets. This is the result of overfitting, which is the tendency of models to capture some of the random variation that is present in any dataset. Overfitting is more problematic when the sample size is insufficient for model development.

Hence, prediction model development studies that use the same data for estimating the developed model’s predictive performance should also include a procedure to estimate optimism corrected performance, known as internal validation. The methods are often referred to as internal validation because no new dataset is being used; rather, performance is estimated internally using the dataset at hand. The most common approaches for internal validation include bootstrapping or cross validation. These methods aim to give more realistic estimates of the performance that we might expect in new participants from the same underlying population that was used for model development.

**External validation studies†**

These studies aim to assess (and compare) the predictive performance of one or more existing prediction models by using participant data that were not used to develop the prediction model. External validation can also be part of a model development study. It involves calculating outcome predictions for each individual in the validation dataset using the original model (ie, the developed model or formula) and comparing the model predictions to the observed outcomes. An external validation can be used for participant data collected by the same investigators, with the same predictor and outcome definitions and measurements but sampled from a later time period; used by other investigators in another hospital or country; used in similar participants, but from an intentionally different setting (eg, models developed in secondary care and tested in similar participants, but selected from primary care); or even in other types of participants (eg, models developed in adults and tested in children, or developed for predicting fatal events and tested for predicting non-fatal events).

Randomly splitting a single dataset (at the participant level) into a development and a validation part is often erroneously referred to as a form of external validation† of the model. In fact, this process is a weak and inefficient form of internal validation: for small datasets, it reduces the development sample size and leaves insufficient data for evaluation, while for large sample sizes, the two parts only differ by chance, and is thus a weak evaluation.

For large datasets, a non-random split (eg, at the hospital level) might be useful—although more informative approaches (eg, internal-external cross validation) are available to examine (heterogeneity) model performance.^7^ A chronological split into development and validation parts resembles temporal external validation, but usually this process is still different to a completely separate validation study conducted at a later point in time. When a model performs poorly, a validation study can be followed by updating or adjusting the existing model (eg, recalibrating or extending the model by including additional predictors).

*TRIPOD=Transparent reporting of a multivariable prediction model for individual prognosis or diagnosis; E&E=explanation and elaboration document.†The term validation, although widely used, is misleading, because it indicates that model validation studies lead to a “yes” (good validation) or “no” (poor validation) answer on the model’s performance. However, the aim of internal or external validation is to evaluate (quantify) the model’s predictive performance.

**Fig 1:**
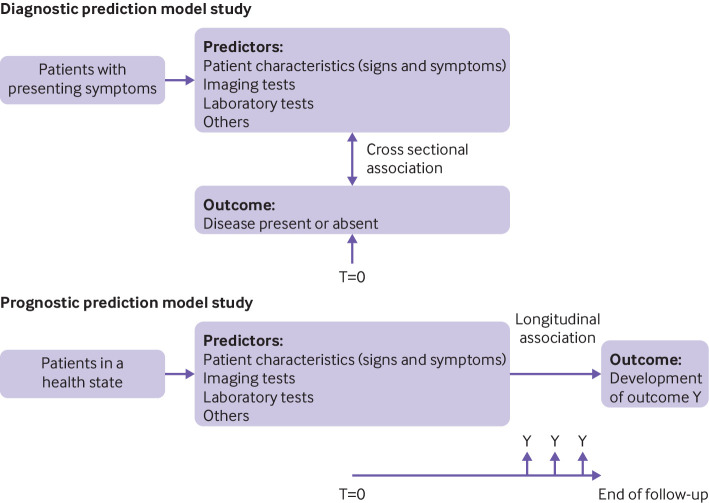
Schematic representation of diagnostic and prognostic prediction modelling studies. T=time of prediction. Adapted by permission BMJ Publishing Group Limited [Transparent reporting of a multivariable prediction model for individual prognosis or diagnosis (TRIPOD): the TRIPOD statement. Collins GS, Reitsma JB, Altman DG, Moons KGM. *BMJ* 2015;350:g7594]^1^

Prediction model studies can look at the development of an entirely new prediction model, or the evaluation of a previously developed model in new data (often referred to as model validation) (box 1). Model development studies also include studies that update the model parameters (or include additional predictors) with the goal of improving the predictive accuracy in a specific setting, for example, in a different subgroup (such as race/ethnic group or sex/gender) or in a specific hospital or country.^11^ ^12^ ^13^

The development and validation of prediction models is often based on participant level data from a specific setting such as a single hospital, institute, or centre. However, datasets might combine or use participant level data from multiple sources or settings (referred to here as clusters). An example is an individual participant data (IPD) meta-analysis that uses IPD from multiple studies or sources, and another is an electronic health records (EHR) study containing IPD recorded from multiple hospitals or general practices.

Although single cluster studies (eg, from one particular hospital) can be convenient (eg, to facilitate data collection), they can result in unwanted problems for either model development or validation.^14^ Firstly, single cluster studies will typically have smaller sample sizes than studies with data from multiple clusters. Prediction models that are developed in small datasets are prone to overfitting and tend to have poor reproducibility.^15^ Briefly, overfitting implies that the model is too much tailored to the development sample, and no longer yields accurate predictions for new individuals from the same source population. To reduce the risk of overfitting, it is generally recommended to adopt penalisation (ie, shrinkage) methods that decrease the variability of model predictions. However, the effectiveness of these methods can be very limited when the sample size is low.^16^

Secondly, estimates of prediction model performance can be very imprecise when derived from small samples.^17^ ^18^ Although it has been suggested that at least 100 events and 100 non-events is recommended when assessing prediction model performance,^19^ even larger samples can give imprecise estimates.^20^ Unfortunately, the sample size of (single cluster) validation studies is often limited.^21^ For example, a systematic review of prediction models for coronavirus disease 2019 found that most (79%) published validation studies included fewer than 100 events.^22^

Thirdly, the intended use of prediction models is rarely restricted to the narrow setting or context from which they were developed. In practice, prediction models are often applied months or even years after their development, possibly in new hospitals, medical settings (eg, from primary to secondary care), domains (eg, from adults to children), regions, or even countries. Although single centre validation studies might help to query the model’s performance in a new sample, they do not directly reveal the model’s extent of transportability and generalisability across different but related source populations.^15^

Fourthly, when combining data from multiple clusters to a single dataset but with multiple data clusters, participants from the same cluster in the datasets (eg, the same hospital, centre, city, or perhaps even country) will often tend to be more similar than participants from different clusters. Participants from the same cluster will have been subject to similar healthcare processes and other related factors. Hence, correlation is likely to be present between observations from the same data cluster.^23^ This effect is also known as clustering, and can lead to differences (or heterogeneity) between clusters regarding participant characteristics, baseline risk, predictor effects, and outcome occurrence. As a consequence, the performance of prediction models developed or validated in clustered datasets will tend to vary across the clusters.^24^ ^25^ ^26^ Using a dataset from a single cluster to develop or validate a prediction model can therefore be of limited value, because the resulting model or model performance is unlikely to generalise to other relevant clusters that intend to use the prediction model.

The TRIPOD (transparent reporting of a multivariable prediction model for individual prognosis or diagnosis) statement was published in 2015 to provide guidance on the reporting of prediction model studies.^1^ ^2^ ^27^ It comprises a checklist of 22 minimum reporting items that should be looked at in all prediction model studies, with translations available in Chinese and Japanese (https://www.tripod-statement.org/). However, the 2015 statement does not detail important issues that arise when prediction model studies are based on clustered datasets. For this reason, we developed a new standalone guideline for prediction model reporting, TRIPOD-Cluster, that provides guidance for transparent reporting of studies that describe the development or validation (including updating) of a diagnostic or prognostic prediction model using clustered data.^28^ TRIPOD-Cluster covers both diagnostic and prognostic prediction models, and we will collectively refer to them more generally as “prediction models” in this article, while highlighting issues that are specific to either type of model.

**Summary points**

To evaluate whether a prediction model is fit for purpose, and to properly assess their quality and any risks of bias, full and transparent reporting of prediction model studies is essential
TRIPOD-Cluster is a new reporting checklist for prediction model studies that are based on clustered datasets
Clustered datasets can be obtained by combining individual participant data from multiple studies, by conducting multicentre studies, or by retrieving individual participant data from registries or datasets with electronic health records; presence of clustering can lead to differences (or heterogeneity) between clusters regarding participant characteristics, baseline risk, predictor effects, and outcome occurrence
Performance of prediction models can vary across clusters, and thereby affect their generalisability
Additional reporting efforts are needed in clustered data to clarify the identification of eligible data sources, data preparation, risk-of-bias assessment, heterogeneity in prediction model parameters, and heterogeneity in prediction model performance estimates

## Types of clustered datasets covered by TRIPOD-Cluster

We distinguish three types of large clustered datasets that are dealt with by TRIPOD-Cluster. For example, IPD could come from:

- Existing multiple studies (eg, cohort studies or randomised trials) where each study contributes separate datasets that are combined into a single dataset (clustering by study)
- Large scale, multicentre studies (which cluster by centre)
- EHR or registries from multiple practices or hospitals (clustering by practice or hospital).

Examples of large, clustered datasets of each type are provided in table 1. A specific type of clustered datasets arises when predictor or outcome variables are assessed at multiple time points.^34^ Because repeated measurements are clustered within individuals, their analysis requires special efforts during the development and validation of prediction models,^34^ ^35^ which is beyond the scope of TRIPOD-Cluster.

**Table 1:** Examples of large datasets with clustering

Dataset	IMPACT^29^	EPIC^30^	CPRD^31^	MIMIC-III^32^
Population	Patients with a head injury	Volunteers agreeing to participate	Patients attending primary care practices in the UK	Patients admitted to the Beth Israel Deaconess Medical Center (Boston, MA, USA)
Data source	IPD from multiple studies	IPD from a prospective multicentre study	Linked database with EHR data	Hospital database with EHR data
Total sample size	11 022	519 978	11 299 221	38 597
No of clusters	15 studies	23 centres	674 general practices	5 critical care units
Heterogeneity in study designs	Phase 3 clinical trials; observational cohort studies	Observational cohort studies; nested case-control studies	Not applicable	Not applicable
Heterogeneity in included populations	Data collection from 1984 to 1997; data from high, low, and middle income countries; variable severity of brain injury	Participant enrolment from 1992 to 2000; data from 10 European countries; heterogeneity in participant recruitment schemes	Data collection from 1987 to present; data from England, Wales, Scotland, and Northern Ireland	Data collection from 2001 to 2012; variable patient ethnic group and social status, among other factors
Heterogeneity in data quality	Variable classification for head injuries; variable time points for outcome assessment	Lack of standardised procedures across cohorts; heterogeneity in dietary assessment methods; heterogeneity in anthropometric measurement methods; heterogeneity in questionnaires across countries	Selective linkage with other databases; large variation in data recording between practices; variable frequency of data recording by age, sex, and underlying morbidity; informative missingness of patient characteristics; non-standardised definitions of diagnoses and outcomes; possible variation in extent of misclassification between diseases	Different critical care information systems in place during data collection; protected health information removed from free text fields
Heterogeneity in level of care	Variability in level of local care; clear improvement of treatment standards over time	Not applicable	Not applicable	Variable efforts to health prevention owing to variability in health insurance programmes among patients^33^

IPD=individual participant data; EHR=electronic health records data; IMPACT=International Mission for Prognosis And Clinical Trial; EPIC=European Prospective Investigation into Cancer and Nutrition; CPRD=Clinical Practice Research Datalink; MIMC-III=Medical Information Mart for Intensive Care III.

### Individual participant data from existing multiple studies

A common approach to increase sample size and capture variability between clusters is to combine the IPD from multiple primary studies.^5^ ^14^ ^36^ ^37^ ^38^ Eligible datasets are ideally, but not necessarily, identified through a systematic review of published primary studies.^39^ Alternatively, datasets can be obtained through a data sharing platform. For instance, the International Mission for Prognosis And Clinical Trial (IMPACT) database was set up in 2007 to combine the IPD from patients with head injuries who participated in different randomised trials and observational studies.^29^ The included studies adopted different protocols, predictor and outcome definitions, measurement times, and data collection procedures. As a consequence, baseline population characteristics differed among the included studies, with variability being particularly high in some of the observational studies.^40^ Furthermore, some trials had lower mortality, possibly because they excluded patients with severe head injury. Using so-called IPD meta-analysis (IPD-MA) techniques, the resulting large IMPACT database has been used to develop various prediction models, for example, to estimate the risk of mortality at six months in patients with traumatic brain injury. Several efforts were made to investigate the presence and potential impact of heterogeneity between clusters (here, between studies) in performance.^40^

As another example, Geersing et al conducted an IPD-MA to assess the performance of the Wells rule, a prediction model for predicting the presence of deep vein thrombosis.^41^ Eligible datasets were identified by contacting principal investigators of published primary studies on the diagnosis of deep vein thrombosis, and by conducting a literature search. Authors of 13 studies provided datasets, after which predictive performance of the Wells rule and its heterogeneity between studies was investigated using meta-analysis techniques.

### Individual participant data from predesigned multicentre studies

An IPD database can also be set up by establishing a collaboration of participating researchers across multiple centres (eg, primary, secondary, or tertiary healthcare practices) by design and thus before data collection. Such predesigned multicentre studies are typically prospective and share a common master protocol outlining participant eligibility criteria, variable definitions, measurement methods, and other study features. Participating investigators and healthcare professionals agree before the start of their study to pool their data, and to predefine the participant eligibility criteria, data collection methods, and analysis techniques. For example, the European Prospective Investigation into Cancer and Nutrition (EPIC) study is a multinational cohort where 23 research centres within Europe with prospectively collected IPD from more than half a million participants to study the role of nutrition in the causation and prevention of cancer.^30^ Questionnaires and interviews were used to retrieve baseline data on diet and non-dietary variables, as well as anthropometric measurements and blood samples. Participant outcomes were determined using insurance records, cancer registries, pathology registries, mortality registries, active follow-up, and death records collection. Over the past few years, the EPIC dataset has been used to develop and validate prognostic risk prediction models for ovarian cancer,^42^ colorectal cancer,^43^ HIV infection,^44^ type 2 diabetes,^45^ and several other disease outcomes.

When combining IPD from existing studies, these studies could be multicentre studies themselves. Typically, however, when such study datasets are used for prediction model development or validation, the data are often considered as a single study dataset. But in this scenario as well, developed and validated models could be subject to variability in predictive accuracy across different centres.

### Electronic healthcare records or registries from multiple practices or hospitals

Another type of clustered data are large databases with routinely collected data from multiple hospitals, primary care, or other healthcare practices.^46^ ^47^ ^48^ These registry databases usually contain EHR for thousands or even millions of individuals from multiple practices, hospitals, or countries. Prediction model studies using EHR data are increasingly common.^48^ For example, QRISK3 was developed using EHR data from 1309 QResearch general practices in England.^49^ Unlike data that are collected specifically for research purposes, EHR data are collected as per routine practice requirements.^48^ As a consequence, data quality and completeness often varies between individuals, clinical domains, geographical regions, and individual databases.^50^ ^51^ ^52^ ^53^ ^54^ For instance, patients with clinically obvious disease might receive less expensive and less invasive investigation than patients with less severe disease that is more difficult to diagnose. Further problems arise when registries cannot be linked reliably for all patients or have very limited follow-up.

## Challenges and opportunities in using clustered datasets for prediction modelling

Without any recognition or adjustment for clustering when developing a prediction model, the estimated model parameters (eg, baseline risk, predictor effects, or weights) and the resulting predicted probabilities could be misleading. For example, when a standard logistic regression, time-to-event or machine learning model is used that ignores the inherent clustering, the final model might yield estimated probabilities that are too close to the overall outcome frequency in the entire study dataset.^55^ The presence of clustering might also affect the transportability of developed prediction models, and the interpretation of validation study results. In particular, clusters can differ in outcome occurrence, in participant characteristics, or even in predictor effects, which could lead to heterogeneity in prediction model performance across clusters and thereby affect its generalisability. This effect is also known as the spectrum effect.^56^ ^57^

For instance, the ability of a model to distinguish between patients with or without the outcome event, which is often measured by the concordance (c) statistic, depends on the homogeneity of the case mix in the entire dataset: the more similar the values of a predictor are in a given set of individuals, the lower the c statistic tends to be.^26^ With case mix, we refer to the distribution of predictor values and other relevant participant or cluster characteristics (such as treatments received), and to the outcome prevalence or incidence. As an example, Steyerberg et al used IPD from 15 studies to validate a prediction model for unfavourable outcome in patients with traumatic brain injury.^40^ They found that case mix variability was particularly high in the four observational studies (as compared with the remaining 11 clinical trials), which also yielded a higher c statistic (as compared with the clinical trials).

### Heterogeneity in outcomes

The performance of a prediction model can also vary according to the outcome prevalence or incidence within a cluster, because the outcome occurrence might not only be determined by predictors that are included in the model, but also by the distribution of other participant or cluster characteristics. Hence, clusters that vary considerably in outcome prevalence or incidence might also differ in case mix.^56^ ^58^ For example, Wilson et al previously developed a prediction model for coronary heart disease. This model has been validated across different time periods and geographical regions with substantial heterogeneity in baseline characteristics (eg, age, sex) and outcome incidence.^10^ Recently, a systematic review found that the model originally developed by Wilson et al overestimates the risk of developing coronary heart disease, and that the extent of miscalibration substantially varies across settings.^59^

### Heterogeneity in design or patient characteristics

Case mix can also vary between studies with major differences in design or eligibility criteria, or even within individual studies. For example, Vergouwe et al used data from a randomised trial to develop a model to predict unfavourable outcome (ie, death, a vegetative state, or severe disability) in patients with traumatic brain injury.^26^ When the developed prediction model’s performance was assessed in the original development dataset, it yielded a c statistic of 0.740 (which was optimism corrected) and an explained variation (ie, Nagelkerke R^2^) of 0.379. However, when the model was externally validated in the data of another trial with similar eligibility criteria, the c statistic increased to 0.779 and R^2^ increased to 0.485. Further analyses indicated that a large part of the higher performance should be attributed to a more heterogeneous case mix.

### Heterogeneity in predictor effects

The predictive performance of a prediction model, when evaluated in different settings, is not only influenced by case mix variation, but also by differences in predictor effects.^15^ ^26^ Heterogeneity in predictor effects could, for instance, arise when predictors are measured differently (eg, using different equipment, assays, or techniques), recorded at different time point (eg, before or after surgery), or quantified differently (eg, using a different cut-off point to define high and low values) across clusters. The magnitude and distribution of measurement error in predictors might also be inconsistent, which can further contribute to heterogeneity in predictor effects.^60^ ^61^ Many other clinical, laboratory, and methodological differences might also exist across clusters, including differences in local healthcare, treatment or management strategies, clinical experience, disease and outcome definitions, or follow-up durations.

To summarise, developing or validating prediction models on data from a single cluster or on a clustered dataset where clustering is ignored does not allow to adequately study and understand the heterogeneity that we can expect between clusters.^15^ To investigate heterogeneity in prediction model performance and to identify underlying causes of such heterogeneity, we need not only studies in which development and validation takes place in multiple clusters, but also the application of analysis techniques that account for such clustering.^15^ ^26^ ^40^ ^46^ ^62^ Such techniques are often referred to as IPD-MA techniques, and typically adopt hierarchical models (eg, random effects) to account for clustering and for heterogeneity between studies.^63^ Accounting for clustering potentially allows researchers to develop prediction models with improved generalisability across different settings and populations,^38^ ^40^ ^64^ to investigate heterogeneity in prediction model performance across multiple clusters, and to assess the generalisability of model predictions across different sources of variation.^15^ ^40^

## TRIPOD-Cluster

### Aim and outline of this document

Prediction modelling studies based on evidently clustered data have statistical complexities that are not explicitly covered in the original TRIPOD reporting guideline but need complete and transparent reporting.^1^ ^2^ As mentioned above, these items include, for example, the presence and handling of systematically missing data within and across clusters, handling of differences in predictor and outcome definitions and measurements across clusters, the (un)availability of specific primary studies, different analysis strategies to account for the presence and modelling of statistical heterogeneity across clusters, and the generalisability and quantification of prediction model performance across clusters.

TRIPOD-Cluster^28^ provides guidance comprising a checklist of 19 items for the reporting of studies describing the development or validation of a multivariable diagnostic or prognostic prediction model using clustered data (table 2). Studies describing an update (eg, adding predictors), or recalibration of a prediction model using clustered data (ie, a type of model development) are also covered by TRIPOD-Cluster.^28^ The aim of this explanation and elaboration document is to describe the guidance, provide the rationale for the reporting items, and give examples of good reporting. TRIPOD-Cluster^28^ is a reporting guideline and does not prescribe how prediction model studies using clustered data should be conducted. However, we do believe that the detailed guidance provided for each item will help researchers and readers for this purpose. We therefore also summarise aspects of good methodological conduct (and the limitations of inferior approaches) to more broadly outline the benefits and implications of developing and validating prediction models using clustered data.

**Table 2:** Checklist of items to include when reporting a study developing or validating a multivariable prediction model using clustered data (TRIPOD-Cluster)

Section/topic	Item No	Description	Page No
**Title and abstract**			
Title	1	Identify the study as developing and/or validating a multivariable prediction model, the target population, and the outcome to be predicted	
Abstract	2	Provide a summary of research objectives, setting, participants, data source, sample size, predictors, outcome, statistical analysis, results, and conclusions*	
**Introduction**			
Background and objectives	3a	Explain the medical context (including whether diagnostic or prognostic) and rationale for developing or validating the prediction model, including references to existing models, and the advantages of the study design*	
	3b	Specify the objectives, including whether the study describes the development or validation of the model*	
**Methods**			
Participants and data	4a	Describe eligibility criteria for participants and datasets*	
	4b	Describe the origin of the data, and how the data were identified, requested, and collected	
Sample size	5	Explain how the sample size was arrived at*	
Outcomes and predictors	6a	Define the outcome that is predicted by the model, including how and when assessed*	
	6b	Define all predictors used in developing or validating the model, including how and when measured*	
Data preparation	7a	Describe how the data were prepared for analysis, including any cleaning, harmonisation, linkage, and quality checks	
	7b	Describe the method for assessing risk of bias and applicability in the individual clusters (eg, using PROBAST)	
	7c	For validation, identify any differences in definition and measurement from the development data (eg, setting, eligibility criteria, outcome, predictors)*	
	7d	Describe how missing data were handled*	
Data analysis	8a	Describe how predictors were handled in the analyses	
	8b	Specify the type of model, all model building procedures (eg, any predictor selection and penalisation), and method for validation*	
	8c	Describe how any heterogeneity across clusters (eg, studies or settings) in model parameter values was handled	
	8d	For validation, describe how the predictions were calculated	
	8e	Specify all measures used to assess model performance (eg, calibration, discrimination, and decision curve analysis) and, if relevant, to compare multiple models	
	8f	Describe how any heterogeneity across clusters (eg, studies or settings) in model performance was handled and quantified	
	8g	Describe any model updating (eg, recalibration) arising from the validation, either overall or for particular populations or settings*	
Sensitivity analysis	9	Describe any planned subgroup or sensitivity analysis—eg, assessing performance according to sources of bias, participant characteristics, setting	
**Results**			
Participants and datasets	10a	Describe the number of clusters and participants from data identified through to data analysed; a flowchart might be helpful*	
	10b	Report the characteristics overall and where applicable for each data source or setting, including the key dates, predictors, treatments received, sample size, number of outcome events, follow-up time, and amount of missing data*	
	10c	For validation, show a comparison with the development data of the distribution of important variables (demographics, predictors, and outcome)	
Risk of bias	11	Report the results of the risk-of-bias assessment in the individual clusters	
Model development and specification	12a	Report the results of any assessments of heterogeneity across clusters that led to subsequent actions during the model’s development (eg, inclusion or exclusion of particular predictors or clusters)	
	12b	Present the final prediction model (ie, all regression coefficients, and model intercept or baseline estimate of the outcome at a given time point) and explain how to use it for predictions in new individuals*	
Model performance	13a	Report performance measures (with uncertainty intervals) for the prediction model, overall and for each cluster	
	13b	Report results of any heterogeneity across clusters in model performance	
Model updating	14	Report the results from any model updating (including the updated model equation and subsequent performance), overall and for each cluster*	
Sensitivity analysis	15	Report results from any subgroup or sensitivity analysis	
**Discussion**			
Interpretation	16a	Give an overall interpretation of the main results, including heterogeneity across clusters in model performance, in the context of the objectives and previous studies*	
	16b	For validation, discuss the results with reference to the model performance in the development data, and in any previous validations	
	16c	Discuss the strengths of the study and any limitations (eg, missing or incomplete data, non-representativeness, data harmonisation problems)	
Implications	17	Discuss the potential use of the model and implications for future research, with specific view to generalisability and applicability of the model across different settings or (sub)populations	
**Other information**			
Supplementary information	18	Provide information about the availability of supplementary resources (eg, study protocol, analysis code, datasets)*	
Funding	19	Give the source of funding and the role of the funders for the present study	

TRIPOD-Cluster=transparent reporting of multivariable prediction models developed or validated using clustered data. A separate version of the checklist is also available in the supplementary materials. *Item text is an adaptation of one or more existing items from the original TRIPOD checklist.

Reporting of all relevant information might not always be feasible, for instance, because of word count limits. In these situations, researchers can summarise the relevant information in a table or figure, and provide additional details in the supplementary material.

### Use of examples

For each item, we aimed to present two examples from published articles; one using an IPD-MA from multiple existing studies or a predesigned study with multiple clusters (type 1 and 2 above), and one using EHR data (type 3). By referencing only two types of examples, we do not suggest that the TRIPOD-Cluster guidance is limited to these two settings. These examples illustrate the information that is recommended to report. Our use of a particular example to illustrate a specific item does not imply that all aspects or items of the study were well conducted and reported, or that the methods being reported are necessarily the best methods to be used in prediction model research. Rather, the examples illustrate a particular aspect of an item that has been well reported in the context of the methods used by the study authors. Some of the quoted examples have been edited for clarity, with text omitted (denoted by . . .), text added (denoted by []), citations removed, or abbreviations spelled out, and some tables have been simplified.

## TRIPOD-Cluster checklist: title and abstract

### Item 1: identify the study as developing and/or validating a multivariable prediction model, the target population, and the outcome to be predicted

The main purpose of this item is to recommend an informative title to help readers easily identify relevant articles. The original TRIPOD guidance recommends that authors should include four main issues in their title^2^; here, we add a fifth item:

- The target population in which the model was developed, validated, or updated
- The outcome to be predicted
- Whether it is a diagnostic or prognostic model (which might already be clear from the target population and outcome to be predicted, or from the prediction model’s acronym)
- Whether the paper reports model development, external validation (including updating), or both
- The type of clustering—for example, IPD from a given number of clusters (eg, studies, hospitals), a multicentre study, or EHR data from a given number of practices or hospitals

The examples given demonstrate that these five aspects can easily be resolved in a title without making it unnecessarily long. For example, the target population sometimes directly indicates whether the model has a diagnostic or a prognostic aim.^1^ If a study internally or externally validates an existing prediction model with a known name or acronym, then this name should be mentioned in the title. The title can also include the type of predictors used, such as the addition of laboratory predictors to an existing prediction model.

#### Example of individual participant data meta-analysis

Pooled individual patient data from five countries were used to derive a clinical prediction rule for coronary artery disease in primary care.^65^

#### Example using electronic health records data

Development and validation of prediction models to estimate risk of primary total hip and knee replacements using data from the UK: two prospective open cohorts using the UK Clinical Practice Research Datalink.^66^

### Item 2: provide a summary of research objectives, setting, participants, data source, sample size, predictors, outcome, statistical analysis, results, and conclusions

This item largely follows the same recommendations and guidance as in the original TRIPOD,^1^ ^2^ and the TRIPOD for Abstracts guidance,^67^ with some additions.

The abstract should provide enough detail to help readers and reviewers identify the study and then persuade them to read the full paper. It should describe the objectives, study design, analysis methods, main findings (such as a description of the model and its performance), and conclusions. Journal word count restrictions will dictate how much of this detail can be presented. For example, it might not be possible to list all potential predictors evaluated for inclusion in the prediction model in a development study.

Ideally, the predictors included in the final model or their categories (eg, sociodemographic predictors, history taking and physical examination items, laboratory or imaging tests, and disease characteristics) can be listed. Abstracts reporting prediction model studies using clustered datasets should also include:

- An explicit reference to whether the study is based on a systematic review of available datasets, a convenient combination of existing datasets, a single multicentre study, or a clustered EHR database
- The level within which individuals are clustered (eg, studies, datasets, countries, regions, centres, hospitals, practices) and number of clusters
- A summary of how the data were used for model development or validation (eg, which clusters were used to develop and validate the model, the use of internal-external cross validation (as explained below))
- How clustering was dealt with in the analysis (eg, one stage IPD-MA)
- Information about the heterogeneity in the model’s performance across clusters.

#### Example of individual participant data meta-analysis 

“Background: External validations and comparisons of prognostic models or scores are a prerequisite for their use in routine clinical care but are lacking in most medical fields including chronic obstructive pulmonary disease (COPD). Our aim was to externally validate and concurrently compare prognostic scores for 3-year all-cause mortality in mostly multimorbid patients with COPD.

“Methods: We relied on 24 cohort studies of the COPD Cohorts Collaborative International Assessment consortium, corresponding to primary, secondary, and tertiary care in Europe, the Americas, and Japan. These studies include globally 15 762 patients with COPD (1,871 deaths and 42 203 person years of follow-up). We used network meta-analysis adapted to multiple score comparison (MSC), following a frequentist two-stage approach; thus, we were able to compare all scores in a single analytical framework accounting for correlations among scores within cohorts. We assessed transitivity, heterogeneity, and inconsistency and provided a performance ranking of the prognostic scores.

“Results: Depending on data availability, between two and nine prognostic scores could be calculated for each cohort. The BODE score (body mass index, airflow obstruction, dyspnea, and exercise capacity) had a median area under the curve (AUC) of 0.679 [1st quartile − 3rd quartile=0.655 − 0.733] across cohorts. The ADO score (age, dyspnea, and airflow obstruction) showed the best performance for predicting mortality (difference AUC_ADO_ − AUC_BODE_=0.015 [95% confidence interval (CI)= −0.002 to 0.032]; P=0.08) followed by the updated BODE (AUC_BODE updated_ − AUC_BODE_=0.008 [95% CI= −0.005 to 0.022]; P=0.23). The assumption of transitivity was not violated. Heterogeneity across direct comparisons was small, and we did not identify any local or global inconsistency.

“Conclusions: Our analyses showed best discriminatory performance for the ADO and updated BODE scores in patients with COPD. A limitation to be addressed in future studies is the extension of MSC network meta-analysis to measures of calibration. MSC network meta-analysis can be applied to prognostic scores in any medical field to identify the best scores, possibly paving the way for stratified medicine, public health, and research.”^68^

#### Example using electronic health records data

“Background. An easy-to-use prediction model for long term renal patient survival based on only four predictors [age, primary renal disease, sex and therapy at 90 days after the start of renal replacement therapy (RRT)] has been developed in The Netherlands. To assess the usability of this model for use in Europe, we externally validated the model in 10 European countries.

“Methods. Data from the European Renal Association European Dialysis and Transplant Association (ERA-EDTA) Registry were used. Ten countries that reported individual patient data to the registry on patients starting RRT in the period 1995–2005 were included. Patients <16 years of age and/or with missing predictor variable data were excluded. The external validation of the prediction model was evaluated for the 10- (primary endpoint), 5- and 3-year survival predictions by assessing the calibration and discrimination outcomes.

“Results. We used a dataset of 136 304 patients from 10 countries. The calibration in the large and calibration plots for 10 deciles of predicted survival probabilities showed average differences of 1.5, 3.2 and 3.4% in observed versus predicted 10-, 5- and 3-year survival, with some small variation on the country level. The c index, indicating the discriminatory power of the model, was 0.71 in the complete ERA-EDTA Registry cohort and varied according to country level between 0.70 and 0.75.

“Conclusions. A prediction model for long term renal patient survival developed in a single country, based on only four easily available variables, has a comparably adequate performance in a wide range of other European countries.”^69^

## TRIPOD-Cluster checklist: introduction

### Item 3a: explain the medical context (including whether diagnostic or prognostic) and rationale for developing or validating the prediction model, including references to existing models, and the advantages of the study design

The original TRIPOD guidance^1^ ^2^ also applies to prediction model studies using clustered datasets. Authors should describe:

- The medical context and target population for which the prediction model is intended (eg, diagnostic model to predict the probability of deep vein thrombosis in patients with a red or swollen leg or a prognostic model to predict the risk of remission in women diagnosed with breast cancer)
- The predicted health outcomes and their relevance
- The context and moment in healthcare when the prediction should be made (eg, predict before surgery the risk of postoperative nausea and vomiting in a patient undergoing surgery, or predict in the second trimester of pregnancy the risk of developing pre-eclampsia later in the pregnancy)
- The consequences or aims of the model predictions (eg, a diagnostic model to guide decisions about further tests or a prognostic model to guide decisions about treatment or preventive interventions)
- If needed, the type of predictors studied (eg, adding specific types of predictors, such as laboratory measurements obtained from more advanced tests, to established, easily obtainable predictors).

Authors of prediction model development studies should reference any existing models and indicate why a new model is needed, ideally supported by a systematic review of existing models.^70^ Authors who validate a prediction model should explicitly reference the original development study and any previous validations of that model and discuss why validation is needed for the current setting or population.

Finally, authors should stress why the study is carried out on a clustered dataset and clarify the clinical relevance of the clusters. For example, IPD from multiple studies might be used during model development to increase sample size, improve the identification and estimation of predictors, or increase generalisability.^14^ ^38^ When analysing large registries with EHR data, the presence of clustering might help to tailor predictions to centres with specific characteristics.^71^ In validation studies, clustered data might be used to evaluate heterogeneity in model performance and to determine whether the model needs to be updated for specific settings or subpopulations.^40^ ^46^ ^72^ When multiple sources of clustering are present (eg, if individuals are clustered by centre and by physician), authors should motivate and report which clusters were chosen for the analysis.

#### Example of individual participant data meta-analysis

“Various clinical decision rules have been developed to improve the clinical investigations for suspected deep vein thrombosis. These rules combine different clinical factors to yield a score, which is then used to estimate the probability of deep vein thrombosis being present. The most widely used clinical decision rule is probably that developed by Wells and colleagues . . . Although the Wells rule seems to be a valid tool in the clinical investigation of suspected deep vein thrombosis in unselected patients, its validity in various clinically important subgroups is unclear; most original diagnostic studies on deep vein thrombosis contained few patients in these important subgroups. To determine whether the Wells rule behaves differently in such subgroups we combined individual patient data from 13 diagnostic studies of patients with suspected deep vein thrombosis (n=10 002). Such meta-analyses of individual patient data (data of individual studies combined at patient level) provide a unique opportunity to perform robust subgroup analyses.”^41^

#### Example using electronic health records data

“It is hypothesised that earlier detection of acute kidney injury (AKI) may improve patient outcomes through the increased opportunity to treat the patient and the prevention of further renal insults in the setting of evolving injury . . . Over the last few years, several groups have reported both electronic health record (EHR)-based and non-EHR-based risk algorithms that can forecast AKI earlier than serum creatinine. Many of these algorithms use patient demographics, past medical history, vital signs, and laboratory values. However, the EHR contains a wealth of other data that could be used to predict the development of AKI, including nephrotoxin exposure, fluid administration, and other orders and treatments. These additional variables could improve model accuracy, resulting in improved detection of AKI and fewer false positives.”^73^

### Item 3b: specify the objectives, including whether the study describes the development or validation of the model

This item remains unchanged from the original TRIPOD guidance.^1^ ^2^ The objectives (typically at the end of the introduction) should summarise in a few sentences the healthcare setting, target population, and study focus (model development, validation, or both). Clarifying the study’s main aim facilitates its critical appraisal by readers.

#### Example of individual participant data meta-analysis

“We therefore analysed clinical, cognitive, and genetic data of patients with amyotrophic lateral sclerosis (ALS) from ALS centers in Europe with a view to predicting a composite survival outcome . . . We aimed to develop and externally validate a prediction model in multiple cohorts.”^74^

#### Example using electronic health records data

“This study validates a machine-learning algorithm, *InSight*, which uses only six vital signs taken directly from the EHR, in the detection and prediction of sepsis, severe sepsis and septic shock in a mixed-ward population at the University of California, San Francisco. We investigate the effects of induced data sparsity on *InSight* performance and compare all results with other scores that are commonly used in the clinical setting for the detection and prediction of sepsis.”^75^

## TRIPOD-Cluster checklist: methods

### Item 4a: describe eligibility criteria for participants and datasets

Readers need a clear description of a study’s eligibility criteria to understand the model’s applicability and generalisability. Prediction model studies that combine IPD from multiple sources often identify relevant studies through collaborative networks or a systematic review of the literature.^37^ Authors should clearly report the eligibility criteria for selecting both the studies or datasets, the individual participants with those studies or datasets, and eligibility at the individual participant level for inclusion in the development or validation of a prediction model. 

Studies might be selected on the basis of their study design (eg, data from randomised trials), study characteristics (eg, a predefined sample size), population characteristics (eg, treatments received), or data availability (eg, availability of particular predictors or outcomes). Prediction model studies that involve multicentre data should report the eligibility criteria for both participants and, when possible, for specific centres (eg, location or sample size). Sometimes, the eligibility criteria of the prediction model study can differ from the eligibility criteria used originally for the included individual studies. For example, some of the identified studies might have targeted particular subpopulations (eg, younger patients) or used eligibility criteria that do not fully match the current study. If the prediction model study therefore excluded certain participants from the included studies, this should be clearly described.

#### Example of individual participant data meta-analysis

“IPD for model validation was identified using the same systematical search in PubMed, EMBASE and the Cochrane Library as described above. Prospective studies were included when recording disease status of pneumonia and clinical signs and symptoms . . . Individual studies were included when containing patients who: (a) were at least 18 years old; (b) presented through self-referral in primary care, ambulatory care or at an emergency department with an acute or worsened cough (28 days of duration) or with a clinical presentation of lower respiratory tract infection; (c) consulted for the first time for this disease episode; (d) were immunocompetent.”^76^

#### Example using electronic health records data

“This study used data from the General Practice Research Database in the United Kingdom which is part of the Clinical Practice Research Datalink (CPRD) . . . People in CPRD have now been linked individually and anonymously to the national registry of hospital admission (Hospital Episode Statistics) and death certificates . . . The main study population consisted of people aged 35–74 years, using the November 2011 version of CPRD and drawn from CPRD practices that participated in the linkages . . . The following persons were excluded: (i) those with cardiovascular disease before the index date or with missing dates, (ii) those prescribed a statin before the index date or with missing dates, (iii) those temporarily registered with the practice.”^77^

### Item 4b: describe the origin of the data, and how the data were identified, requested, and collected

Prediction model studies based on existing data sources should fully describe how they identified, requested, and collected that data. Item 7a covers data cleaning, data harmonisation, and database linkage. The provenance of all included data sources should be made clear (eg, citations or web links).

Investigators conducting prediction model studies based on IPD from multiple studies might identify and collect source datasets by performing a review of the literature (systematic or not) and requesting data from the authors or by forming a collaborative network.^37^ If IPD collection from all the eligible studies identified in a review is not possible or not necessary, as is often the case, the reasons for excluding any identified studies should be reported. Prediction model studies that used a systematic review should cite that review if it has been published or should report the full search string, with dates and databases searched, using the PRISMA (preferred reporting items for systematic reviews and meta-analyses)-Search extension (https://osf.io/ygn9w/).

Studies using EHR data must report the data source or registry (eg, by referring to a publication or web link) and data extraction methods (eg, queries relating to structured query language). For prospective multicentre studies, this reporting item mostly relates to data collection procedures such as the use of data storage platforms and encryption standards.

#### Example of individual participant data meta-analysis

“A systematic search of literature was performed to identify all published studies with no restriction on language. This study protocol was started in December 2008, followed by literature searched in PubMed/Medline, Ovid, Web of knowledge and Embase, with additional MESH and free text terms for ‘secondary cytoreductive surgery and ovarian cancer or secondary cytoreductive surgery and ovarian carcinoma’, were supplemented by hand searches of conference proceedings, reference lists in the publications and review articles. We sent the invitation letters to all available investigators or groups of studies we identified, who had reported articles with regard to SCR [secondary cytoreductive surgery]. An international collaborative study group was then set up . . . Individual patient data were collected from all participating groups in which these involved already complete datasets.”^78^

#### Example using electronic health records data

“We gathered data from the cost-accounting systems of 433 hospitals that participated in the Premier Inc Data Warehouse (PDW; a voluntary, fee-supported database) between January 1, 2009, and June 30, 2011 . . . PDW includes ≈15% to 20% of all US hospitalizations. Participating hospitals are drawn from all regions of the United States, with greater representation from urban and southern hospitals.”^79^

### Item 5: explain how the sample size was arrived at

One of the main reasons for using large or clustered datasets to develop or validate a model is the greater sample size and access to a broader population.^14^ ^46^ ^71^ The larger the sample size the better, because large samples lead to more precise results. The effective sample size in a prediction model study is calculated differently depending on the type of outcome:

- Binary outcomes: the smaller of the two outcome frequencies^80^ ^81^ ^82^
- Time-to-event outcomes: the number of participants with the event by the main time point of interest^80^
- Continuous outcomes: the number of participants.^81^ ^83^

#### Sample size considerations for model development

Prediction models developed using small datasets are likely to be affected by overfitting, particularly if they have many candidate predictors relative to the number of outcome events.^84^ Empirical simulations focusing on accuracy and precision of the regression coefficients (rather than model performance) have suggested that at least 10 participants with the outcome event per variable are needed.^85^ More precisely, this value is the number of parameters (degrees of freedom) needed to represent these candidate predictors. For example, more than one degree of freedom is required for categorical predictors with two or more levels, and for continuous predictors modelled using splines or fractional polynomials.

However, some researchers have argued that an event per variable of 10 is too conservative 86 and others have suggested larger values of events per variable to avoid bias in estimated regression coefficients.^87^ ^88^ Simulation studies have shown that the presence of clustering and between-study heterogeneity does not affect the sample size needed much for prediction model development; rather, variable selection and the total number of events and non-events are more influential.^89^ Recent simulation studies have shown no rationale for the rule of thumb regarding 10 events per variable,^90^ and no strong relation between event per variable and predictive performance.^91^

These recent results suggest that sample size requirements should be tailored to the problem and setting. Minimum sample size criteria have been proposed for models developed using linear regression,^83^ logistic,^80^ ^91^ and time-to-event models.^80^ Adaptive sample size procedures have also been proposed.^92^ Models developed using internal-external cross validation (see below) should ensure that each omitted cluster is sufficiently large for validation. Although formal guidance about the minimal number of clusters or minimum sample size per cluster is currently lacking, the fact that the effective sample size of clustered datasets decreases as the similarity of study participants within each cluster increases is well known.^93^

Authors should explain how the sample size was determined, fully describing any statistical or practical considerations, including any estimates (rationale and provenance) used in the calculation. Sample size is often determined by practical considerations, such as time, data availability (particularly when obtaining IPD from multiple studies), and cost. In these instances, it is helpful to discuss the adequacy of the sample size in relation to the number of predictors under study, the need for variable selection, and the primary performance measures.

#### Sample size considerations for model validation

Validation studies aim to quantify a model’s predictive performance when evaluated on a different dataset to that used in development. Current recommendations suggest that at least 100 participants with the outcome and 100 without the outcome are needed,^19^ while more than 200 outcome events are preferable to ensure precise estimates of predictive performance.^20^ ^82^ ^94^ ^95^ Datasets tend to be large when originating from multiple sources or EHR. Small sample concerns are then less of an issue, unless the outcome is very rare or model performance is evaluated in each dataset or cluster.^46^ Authors should explain how they determined the sample size and whether they considered the potential presence of clustering.

If investigators use clustered data, they should highlight which clusters they used to develop the model and which to validate the model. Sample size requirements should be considered for both model development and validation, which might affect which datasets or clusters are used. For example, if a single cluster is kept back for validation, it must contain enough outcome events to be useful for evaluating predictive performance and obtaining precise estimates. However, if a very large cluster is kept from model development to use in validation, the resulting model might have less accurate predictions (ie, more overfitting concerns) especially if the clusters used for development are much smaller. In this scenario, it would be preferable to use all data for model development.

#### Internal-external cross validation

**Fig 2:**
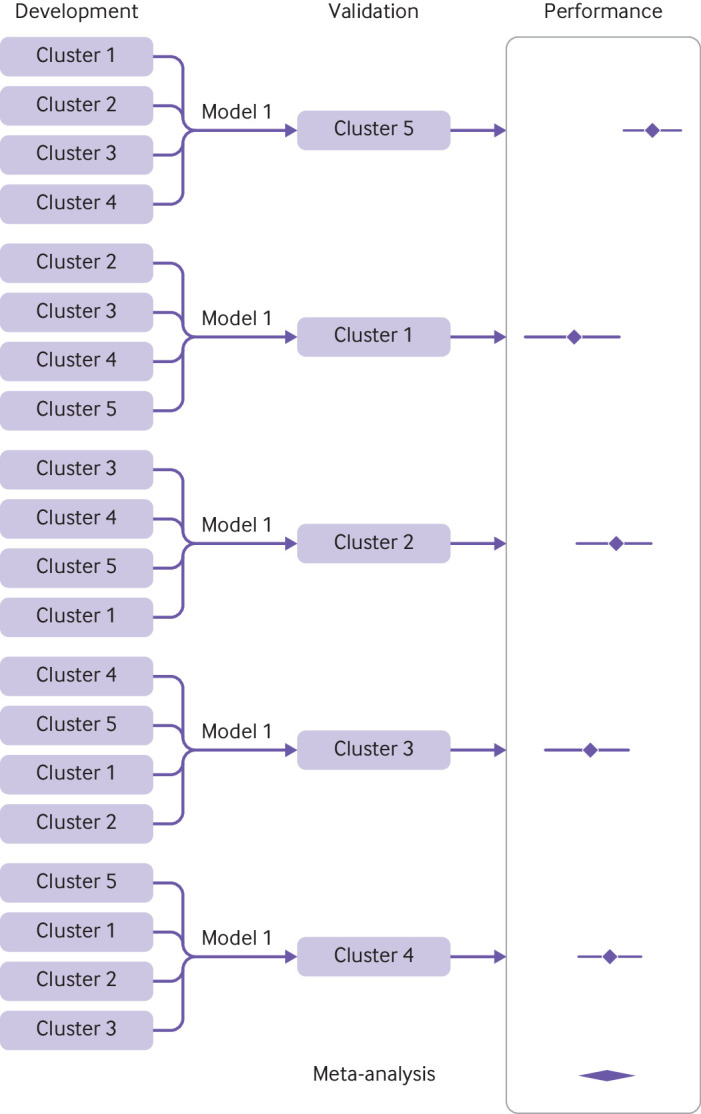
Illustration of internal-external cross validation

Internal-external cross validation combines the strength of external validation with the strength of prediction model development using all available data.^7^ ^96^ Here, a model is developed using the full data minus one cluster, then validating this model using the excluded cluster (fig 2). This process is repeated so that each cluster is omitted and reserved for validation in turn. The consistency of the developed model and its performance can then be examined on multiple occasions. Heterogeneity in performance can then be examined across the different settings and populations represented by the clusters (see also item 8f).

For example, Takada et al implemented internal-external cross validation to evaluate different modelling strategies for predicting heart failure.^97^ To this purpose, they used an existing large population level dataset that links three sources of EHR databases in England: primary care records from the Clinical Practice Research Datalink, secondary care diagnoses and procedures recorded during admissions in Hospital Episodes Statistics, and the cause specific death registration information sourced from the Office for National Statistics registry. This clustered dataset included 871 687 individuals from 225 general practices, and was used to assess the model’s discrimination and calibration performance across the included practices.

#### Example of individual participant data meta-analysis

“A general rule of thumb is for at least 10 events to be available for each candidate predictor considered in a prognostic model. There were seven candidate predictors (age, sex, site of VTE [venous thromboembolism], BMI [body mass index], D-dimer post-treatment, lag time and treatment duration) for consideration, but some of these were continuous predictors, which may potentially require non-linear modelling (e.g., fractional polynomials) that would slightly increase the number of variables further (e.g., if age+age^2^ is included, then “age” relates to two predictors). The RVTEC database has seven trials in total, with 1,634 patients with follow-up information post-treatment and 230 of these have a recurrence . . . six of the seven trials are used for model development, so there are between 1,196 and 1,543 patients and between 161 and 221 recurrences available for the development phase of the prognostic models. Thus, there will be at least 23 (=161 events divided by seven candidate predictors) events for each of the seven candidate predictors, which is considerably greater than the minimum 10 per variable required, this gives adequate scope for fractional polynomial modelling of non-linear trends as necessary . . . Furthermore, the external validation databases also had large numbers. The RIETE database has 6,291 patients with follow-up information post-treatment and 742 of these have a recurrence.”^98^

#### Example using electronic health records data

“Based on a previous work using linked CPRD data in which 5118 incident cirrhosis patients were identified in primary or secondary care during a 12-year period from 1998 to 2009, we estimate that at least 426 patients per year are diagnosed with cirrhosis. Therefore, during our study period from 1998 to 2016, there will be around 6887 new cirrhosis cases (study period ends in February 2016) . . . This will give us a population of approximately 765 111 patients (with an abnormal liver blood test result) with the estimated 3443 outcome events. As we are planning to omit 30 GP practices for model validation, assuming all of the 411 practices will be included in our dataset and an even distribution of outcome events across practices, we will have 251 events in the validation dataset (above the estimated minimum requirement of 100 events). For our development dataset, we will have 3192 events. We are interested in 39 predictor variables that may require potentially as many as 65 parameters to be estimated (counting in multiple categories and assuming each of our 14 continuous predictors requires an extra parameter for a non-linear term). This would provide our model with 49 events per variable.”^99^

### Item 6a: define the outcome that is predicted by the model, including how and when assessed

A clear definition and description of how and when the outcomes are measured is key. Readers need this information to judge what the actual outcome was and whether any outcomes were missed or misclassified. The original TRIPOD guidance and explanation and elaboration document (E&E) give clear explanations of how to report outcome assessment and the differences in diagnostic and prognostic prediction studies,^2^ which we do not repeat here.

Multiple data sources or clusters might have defined or measured their common outcomes slightly differently (see also item 7b). Authors should clearly report these differences and any efforts to redefine or reclassify outcomes from particular data sources to a common outcome definition and classification (see also item 7a).

Prediction model studies using registry data could be at higher risk for problems in detecting or classifying the outcome than when data from dedicated, prospective studies are used. Authors are encouraged, where possible, to report each source study’s protocol for measuring the outcome, data checks, and quality measures.

#### Example of individual participant data meta-analysis

“The outcome was time to death in years. Participants were contacted by study interviewers in every wave and those who were not located or whose relatives informed they had died, had their mortality information confirmed by the national vital statistics records or by a next-of-kin.”^100^

#### Example using electronic health records data

“We defined the primary outcome of critical illness during hospitalization as intensive care unit location stated in the EHRs with concomitant delivery of organ support (either mechanical ventilation or vasopressor use). The delivery of mechanical ventilation was identified using intubation, extubation, and tracheostomy events and ventilator mode data in the EHRs. Vasopressor use was defined as the administration of vasoactive agents (e.g., norepinephrine, dopamine, epinephrine) by infusion for more than 1 h recorded in the EHRs.”^101^

### Item 6b: define all predictors used in developing or validating the model, including how and when measured

Prediction models typically use multiple predictors in combination and might therefore include demographic characteristics, medical history and physical examination items, information on treatments received, and more complex measurements from, for example, medical imaging, electrophysiology, pathology, and biomarkers. When prediction models are developed using clustered data, they might also include characteristics of the healthcare setting (eg, line of care, location). Authors should clearly report how predictors were measured and when, to help readers identify situations where the model is suitable for use.

Authors should indicate whether predictors were measured differently in each cluster and whether any formal harmonisation was done (see also item 7a). Item 7d discusses how to report when one predictor of interest is completely missing from one or more clusters.

Some predictors might be measured differently, for example in EHR datasets and prospective studies, including trials.^102^ For instance, different healthcare providers and systems can record substantially different amounts of detail about medical histories, clinical variables, and laboratory results.^103^ These differences can lead to differences in the measurement error (either random or systematic error), which will affect an existing model’s performance and a new model’s regression coefficients.^60^ ^104^ ^105^ ^106^ Authors should therefore explain how predictors were measured, so that readers can evaluate this information before choosing to use a model in practice.

#### Example of individual participant data meta-analysis

“In all cohorts, information on age, gender, comorbidities, lifestyle factors, and functional status were collected through structured interviews . . . Comorbidities and lifestyle factors included disease history (heart disease, lung disease, stroke, cancer, diabetes, and hypertension), depression, body mass index (BMI), alcohol use, smoking, and physical activity . . . Depression was defined by the Center for Epidemiologic Studies Depression Scale score ≥3 in ELSA, HRS, and MHAS; by 15-item Geriatric Depression Scale score ≥5 in SABE; and by EURO-D score ≥4 in SHARE. Height and body weight were self-reported in all cohorts, except SABE in which both were measured during a visit . . . Participants were considered physically active if they had engaged in vigorous physical activity (sports, heavy housework, or a job that involves physical labour) at least three times a week in HRS, MHAS, and SABE; and at least once a week in ELSA and SHARE.”^100^

#### Example using electronic health records data

Clinical and administrative data were extracted from SingHealth’s electronic health records system, Electronic Health Intelligence System, which is an enterprise data repository that integrates information from multiple sources, including administrative, clinical and ancillary . . . Patient demographics included age, gender, and ethnicity. Social determinants of health included the requirement of financial assistance using Medifund and admission to a subsidized hospital ward . . . For medical comorbidities, chronic diseases such as heart failure, chronic obstructive pulmonary disease, cerebrovascular accident, peripheral vascular disease among other major diseases listed under the Charlson Comorbidity Index, Elixhauser comorbidities and Singapore Ministry of Health Chronic Diseases Program were extracted. These diseases were extracted using International Classification of Diseases 10 codes of primary and secondary discharge diagnoses dating back to seven years.”^107^

### Item 7a: describe how the data were prepared for analysis, including any cleaning, harmonisation, linkage, and quality checks

This new item helps to ensure transparency in how researchers clean, harmonise, and link their obtained data. This information will allow readers to better appraise the quality and integrity of data and determine whether the data are truly comparable and combinable across clusters.

Data cleaning is an essential part of any research study and involves tasks such as identifying duplicate records, checking outliers, and dealing with missing values. The quality of large, routinely collected health data has often been criticised, particularly with respect to their completeness and accuracy.^47^ ^108^ For example, the quality of routinely collected primary care data can vary substantially, because data are entered by general practitioners during routine consultations, and not for the purpose of research.^31^ Researchers must therefore undertake comprehensive data quality checks before undertaking a study. Particular weaknesses include missing data, and the potential for data to be missing not at random; non-standardised definitions of diagnoses and outcomes; interpreting the absence of a disease or outcome recording as absence of the disease or outcome itself, when patients with the disease or outcome can sometimes simply fail to present to the general practitioner; incomplete follow-up times and event dates (such as hospital admission and length of stay); and lack of recording of potentially important predictors.

When IPD are obtained from multiple studies or through a multicentre collaboration, researchers need a careful, often prolonged, process to clean each received dataset and harmonise information, so that the IPD can be combined in a meta-analysis.^39^ ^102^ This process often involves merging the data into one storage/query system (technical harmonisation) and integrating datasets into a logically coherent entity (semantic harmonisation).^109^ Data integration is often achieved by adopting common vocabularies (eg, CDISC Operational Data Model) or taxonomies (eg, SNOMED Clinical Terms) for naming variables or for standardising values for those variables used commonly in clinical research. For example, measurement units can differ across clusters (eg, kilograms *v* pounds for weight) and might therefore require standardisation. In addition, when mapping data from different data sources, we recommend distinguishing between what is measured (eg, systolic blood pressure) and how the measurement is done (eg, using a sphygmomanometer). Authors should describe any process for querying and confirming data with the original investigators, and report any process taken to standardise measurements. The complexities involved in managing IPD can be enormously labour intensive and require considerable clinical insight.^29^ Problems might include identification of the same individual in multiple studies (duplicates), exclusion of ineligible participants who do not meet the inclusion criteria, inconsistent recording of continuous predictors and outcomes between studies, inconsistent timing and method of measuring predictors, and coding of censoring information. Authors should clearly describe how they handled these problems and add any detailed descriptions that do not fit within article word limits to the supplementary material.

Prediction model studies that use routinely collected data should also describe any required data linkage, for example, to join information about a participant’s characteristics from one database to their recorded outcomes in another database (eg, national death registries or even another database from the same cluster). The RECORD (reporting of studies conducted using observational routinely collected data) statement specifically asks researchers to report whether the study “included person-level, institutional-level, or other data linkage across two or more databases,” and, if so, that the study included “the methods of linkage and methods of linkage quality evaluation.”^110^ A flow diagram might be helpful to demonstrate the linkage process.

#### Example of individual participant data meta-analysis

“The final merged dataset, individual formatted files and documentation of all transformations made, were securely transferred to a web-based server at Centro Rosarino de Estudios Perinatales, Rosario, Argentina, a WHO Collaborative Centre in Child and Maternal Health . . . At the study level, we extracted data on the providing collaborator, study design, data source, study period, and study inclusion and exclusion criteria. At the participant level, we extracted information on individual participant characteristics and outcome data as specified in Table 1 . . . Any continuous data for parity were therefore also transformed to the binary form. We however retained the continuous data for any relevant analysis. Assumptions were made in harmonising the ethnicity variable, and this was recoded as White, Black, Asian, Hispanic, Mixed and Other. Pre-gestational diabetes, Type 1 and Type 2 diabetes were harmonised as history of diabetes, while a history of systemic lupus, multiple sclerosis, idiopathic thrombocytopenia, rheumatoid arthritis or antiphospholipid syndrome were harmonised as history of autoimmune disease. We also harmonised history of glomerulonephritis, nephrotic syndrome, nephritis or nephropathy as history of renal disease. Range and consistency checks were carried out on all datasets received and summary tables were produced. Missing data over 10% for each variable, range checks for continuous variables measures, obvious errors, and inconsistencies between pre-identified variables or outlying values were queried and rectified with input from the original authors. Two reminders were sent to the original author to respond to queries, and if no response was received, a decision to exclude the variable in question was made by the project team.”^111^

#### Example using electronic health records data

“We used data from the Clinical Practice Research Datalink covering a representative sample of 7% of the UK general population . . . We selected patients with a recorded consultation for hip pain/osteoarthritis or knee pain/osteoarthritis pain between 1 January 1992 and 31 December 2015 and who had complete registration and no recorded hip pain/osteoarthritis or knee pain/osteoarthritis consultations in the 3 years before their index consultation (the first consultation for hip or knee pain/osteoarthritis recorded in the study period, reflecting a first or new episode). The entry date for each patient was the date of index consultation . . . We used the earliest recorded date of the outcome after the index consultation . . . We excluded patients with hip or knee replacements within 2 years of the index consultation, and those whose records were censored (died, de-registration with practice, or last upload of computerised data) during this 2-year period . . . For missing values in the categorical variables (ethnicity, smoking status, drinking status, mental health disorder, and joint specific osteoarthritis), a “not recorded” category was introduced and combined with the reference category for each variable.”^66^

### Item 7b: describe the method for assessing risk of bias and applicability in the individual clusters (eg, using PROBAST)

For studies based on IPD of multiple source studies or on clustered EHR datasets, researchers face a risk of encountering differences and shortcomings in:

- inclusion of consecutive or non-consecutive participants
- inclusion and exclusion criteria
- definitions of the predictors and outcomes
- how, and how accurately, these predictors and outcomes were measured
- which predictors were collected, as relevant predictors are not always measured in all datasets
- follow-up time for prognostic model studies
- loss to follow-up for prognostic model studies
- number and pattern of missing values.

All these differences can affect the analysis and interpretation of the combined data. The quality and suitability of each individual data source for the research question should be critically appraised by the research team to determine whether risk of bias in that cluster or study could compromise the validity of the eventual results.^14^ ^46^ ^112^

Authors should specify in the methods section whether they used a risk-of-bias method or tool, how they used it, and how they used the findings from the quality assessment in their statistical analysis and in interpreting their study findings. For example, authors might plan sensitivity analyses limited to studies with low overall risk of bias or low risk in particular domains, or might investigate heterogeneity between data sources using subgroups based on risk of bias ratings.^14^ ^46^ Risk of bias and applicability should ideally be assessed separately for each cluster. However, if the number of clusters is very large, the overall quality of the combined dataset can be assessed instead (eg, in a nationwide EHR or registry).

We recommend the use of PROBAST (diagnostic and prognostic prediction model risk of bias assessment tool; www.probast.org), which was developed to assess quality, risk of bias and concerns about applicability in primary prediction model studies.^113^ ^114^ This tool uses signalling questions to decide whether a study has high or low risk of bias in four domains (box 2).

Box 2 **Use of PROBAST domains to rate risk of bias in primary studies**

The PROBAST (diagnostic and prognostic prediction model risk of bias assessment) tool can be used to identify areas where poor quality and bias might be introduced into the prediction model study or where concerns regarding applicability could exist.^113^ ^114^ Use of this tool involves assessment of four domains to cover key aspects of prediction model studies: participants, predictors, outcome, and analysis.

- The domain of participant selection covers potential sources of poor quality, bias, and applicability concerns related to participant selection methods and data sources. It considers whether appropriate data sources were used and whether participants in the included sources or studies were appropriately included or excluded from the prediction model study.
- The predictors domain covers potential sources of poor quality, bias, and applicability concerns related to the definition and measurement of predictors evaluated for inclusion in the model. For instance, it considers any actions to blind assessment of predictors for the outcome and other predictors, whether predictors were defined and assessed in a similar way for all participants in the included data sources or studies, and whether all predictors are available at the time the prediction model is intended to be used.
- The outcome domain covers potential sources of poor quality, bias, and applicability concerns related to the definition and measurement of the outcome predicted by the model. For instance, it considers any actions to blind outcome assessment, whether the outcome in the included data sources or studies was determined appropriately, whether a prespecified or standard outcome definition was used, whether predictors were excluded from the outcome definition, whether the outcome was defined and determined in a similar way for all participants in the data source or study, and whether the time interval between predictor assessment and outcome determination was appropriate.
- The analysis domain covers potential sources of poor quality, bias, in the statistical analysis methods. It is less applicable for the quality appraisal of studies or data sources included in an individual participant data meta-analysis, registry based study, or study based on electronic health records, because the analysis methods in the primary studies typically do not affect the prediction model study.

By contrast, prospective multicentre studies can address quality, risk of bias, and applicability issues during their design. Although it may be illogical to formally use a risk of bias tool for such studies, unexpected events can occur during the data collection phase. It is, for instance, possible that drop-out rates are much higher than anticipated for some of the clusters. These events may affect bias and applicability, and should clearly be described. Also, when an existing multi-centre study is reused for prediction model development or validation but this was not an original aim, formal risk of bias assessments as described above should be considered.

#### Example of individual participant data meta-analysis

“Two independent reviewers assessed the quality of each IPPIC-UK dataset using a modified version of the PROBAST (Prediction study Risk of Bias Assessment) tool. The tool assesses the quality of datasets and individual studies across three domains: participant selection, predictors and outcomes. We classified the risk of bias to be low, high or unclear for each of the relevant domains. Each domain includes signalling questions that are rated as “*yes*,” “*probably yes*,” “*probably no*,” “*no*” or “*no information*.” Any signalling question rated as “*probably no*” or “*no*” indicates a potential for bias in the IPD received for that study, which is therefore classed as having a high risk of bias in that domain. The overall risk of bias of an IPD dataset was considered to be low if it scored low in all domains; high if any one domain had a high risk of bias; and unclear for any other classifications.”^115^

### Item 7c: for validation, identify any differences in definition and measurement from the development data (eg, setting, eligibility criteria, outcome, predictors)

Differences in the setting, eligibility criteria, outcome, and predictors of the validation and development datasets can affect the model’s predictive performance and transportability from the development setting to the validation setting (see also items 10b and 10c). Authors of external validation studies that evaluate the performance of an existing prediction model using clustered data should clearly identify any such differences, as is done when using one data source (item 12 of the original TRIPOD statement). Differences can also occur between the contributing clusters, leading to heterogeneity in prediction model performance. For example, some source studies could have categorised continuous predictors during data collection or could have used different outcome definitions.

The differences between the development and validation data can be intentional, to evaluate whether the model has good predictive performance in different scenarios (eg, different setting, eligibility criteria, or different definitions of outcome or predictors). These intentional differences should then be clearly described. Alternatively, researchers should also specifically state if no differences exist in the healthcare setting, eligibility criteria, outcome, and predictors.

#### Example of individual participant data meta-analysis 

“The cohorts varied greatly in terms of geographic location, sample size, and number of events and included a broad spectrum of patients with COPD [chronic obstructive pulmonary disease] from primary, secondary, and tertiary care settings. Mean forced expiratory volume in 1 s percentage ranged from 30 to 70% of the predicted values, mean modified Medical Research Council dyspnea scores from 1.0 to 2.8 (the scale goes from 0 to 4, with 4 being the worst), mean number of exacerbations in the previous year (where available) from 0.2 to 1.7, and mean 6-min walk distance (where available) from 218 to 487m.”^68^

#### Example using electronic health records data

“In our derivation cohort from England, we analysed information on 321 415 women with 433 353 delivery episodes that resulted in live births or stillbirths with a complete six weeks of post-delivery follow-up. Our validated Swedish cohort had information on 498 918 women with 662 387 deliveries. Table 1 summarises the basic characteristics of the study population. Broadly, women in both cohorts had similar pre-pregnancy body mass index, delivery age, and prevalence of comorbidities (with the exception of varicose veins). Compared with England, women in Sweden were less likely to smoke and had fewer delivery related complications.”^116^

### Item 7d: describe how missing data were handled

Missing values arise when predictor or outcome variables were not fully recorded in primary studies or were measured inconsistently across studies (eg, when a continuous variable was measured on a categorical scale in some studies).^14^ The original TRIPOD E&E describes good practice and key recommendations for dealing with missing values,^2^ which generally consider the use of multiple imputation methods. Multiple imputation aims to allow for uncertainty about missing values by generating multiple copies of the dataset with the missing values replaced by imputed values.^117^ Each imputed dataset is analysed separately using standard statistical methods, and the results combined using Rubin’s rules.^118^

Imputing missing values in heterogeneous data sources has some challenges. Some variables might not be available in one or more clusters, making them systematically missing for all participants in these clusters.^119^ ^120^ Researchers should assess whether these systematically missing variables should be imputed and report whether they made this assessment and how. Important cluster level variables (eg, geographical location of the centre or hospital) can be used during imputation. These variables should be clearly reported (eg, in a table) with reasons for their use and potential missingness. Imputation should account for clustering of participants within studies or centres,^121^ ^122^ to ensure that imputed datasets remain compatible with the intended analyses.^123^ Researchers can impute data in each cluster separately or in all clusters using a hierarchical model.^124^ Advanced imputation methods can deal with a mixture of sporadically and systematically missing variables at the participant level and cluster level.^120^ ^125^

Additional challenges might arise when applying a developed prediction model in routine clinical practice.^126^ ^127^ ^128^ ^129^ ^130^ Many prediction models do not have a direct, built-in problem solving ability in case a predictor value of the individual is missing. Although imputation methods could be used to recover the missing values in real time, their implementation usually requires access to raw data from multiple individuals or access to dedicated imputation models.^127^

Authors of prediction model studies should describe any missing data and how they were handled. A clear distinction should be made between handling of missing values at model development time, at model validation time,^130^ and at model deployment time.^129^ Authors should state if they excluded individuals with missing values from the analysis and explain why.

Box 3 lists the key details that authors should report about how they handled missing data, based on existing guidance.^1^ ^131^ Any details that cannot be reported in the main manuscript owing to word count restrictions should be added to the supplementary information.

Box 3 **Key information to report on handling missing data according to TRIPOD-Cluster**

**In the methods section**

- Possible reasons for any missingness
- A clear description of the method used to account for missing data in the predictors and outcome (eg, complete case, mean imputation, or multiple imputation), with justification
- For analyses based on single or multiple imputation:
  - Provide details of the software used, including any specific imputation routines (eg, ICE, MICE, PROC MI, Amelia, micemd, or aregImpute)
  - List the variables that were included in the imputation procedure, how they were chosen, and whether the outcome was included for imputing the predictors and vice versa
  - Explain how the model accounted for differences in design features (eg, potential heterogeneity between clusters in the predictor-outcome associations)
  - Explain how the imputation model accounted for continuous, binary, categorical, and transformed variables (eg, interaction terms and non-linear terms)
  - Explain how systematically missing variables (if present) were dealt with
  - Report the number of imputations
  - Discuss how convergence was monitored, including any checks of the imputed data (eg, comparing distributions of imputed values with the complete data)
  - Explain how the estimates from each imputed dataset were combined or pooled to get the final estimates.

**In the results section (or supplementary material)**

- The number of missing values per predictor and outcome variable
- The number of missing values per participant using a frequency table
- For analyses based on single or multiple imputation:
  - Diagnostic checks of the imputation model, such as convergence statistics
  - Comparison of the observed and imputed data
- Compare the characteristics of individuals with any missing value and with completely observed data; this comparison will suggest whether certain predictors or outcomes were indeed missing completely at random or whether their missingness was related to observed characteristics.

#### Example of individual participant data meta-analysis

“If a predictor from a prediction model is not present within an individual study (ie, not recorded for any of the participants in that study), this is considered to be systematically missing. Though it may be possible to impute values for the missing predictor based on the IPD from other studies, for practical reasons, imputation will not be performed for systematically missing variables . . . If some participants are missing values for predictors within an individual study, multiple imputations will be used to recover data rather than dropping these participants from the analysis as in a complete case analysis. The multiple imputations will be based on the individual study, not the collection of all IPD studies. The imputation process will be performed before any of the analysis takes place, therefore all relevant predictors (for all prediction models to be validated) will be identified and imputed for at the same time to avoid imputing values for each different prediction model separately. This will ensure a coherent set of imputed datasets, to be used consistently in all analyses, regardless of the prediction model being validated. The interest here is performance statistics, which is sensitive to the type of imputation model. The imputation model will therefore include other variables available within the dataset . . . For each validated model, performance statistics (discussed later) will be averaged across imputations using Rubin’s rules to obtain one estimate and standard error (SE) for each performance statistic in each study. This will be done on the logit scale for the C-statistic, as it is unlikely to be normally distributed on the original scale. Within-imputation SEs can be obtained on the transformed scale by applying the delta-method and using the formulae given by Debray *et al*.”^132^

#### Example using electronic health records data

“When pre-operative grade was missing it was multiply imputed on the basis of post-operative grade . . . Lymph node metastases outcomes in patients who did not undergo lymphadenectomy were multiply imputed assuming missingness not at random (MNAR) and missingness at random, and results obtained using imputed data were compared with those of a complete case analysis in which women who did not undergo lymphadenectomy were excluded (sensitivity analysis). The mice algorithm in R was used, with 60 imputations, to account for the 57% of incomplete cases. The numbers of missing biopsy data and patients without lymphadenectomy are reported in Table 1. All other results are based on multiple imputation under MNAR. Details on the imputation process and results of sensitivity analyses are presented in the appendix and in Supplementary Tables 2 and 3.”^133^

### Item 8a: describe how predictors were handled in the analyses

Researchers must decide how to handle any continuous variables when developing a prediction model. The common practice of converting continuous predictors into categorical predictors should ideally be avoided, because it is biologically implausible and results in considerable loss of predictive performance.^134^ If continuous variables were categorised, authors should explain why, the cut-off points used, and how they were chosen. They should also report any transformations of the continuous variables and how this was done (eg, using fractional polynomials or restricted cubic splines). Authors should make clear if they handled or explored continuous variables separately for each data source and explain how they combined variables or handled them across the separate data sources.

#### Example of individual participant data meta-analysis

“A linear regression was used with the natural logarithm of fat-free mass as the outcome, and weight, height, age, sex, and ethnic group as candidate predictors (variables). Using a stepwise approach through backwards elimination, beginning with a model that included all predictors, we excluded candidate predictors from the saturated model based on their statistical significance (Wald test >0.05). Non-linear relations between outcome and continuous predictors were considered by identifying, at each iterative step of the stepwise process, the best fitting fractional polynomial terms (using Stata command *mfp*) . . . Although heterogeneity and clustering of patients across or within studies was not considered for model development, we checked the impact of this using an internal-external validation approach.”^135^

#### Example using electronic health records data

“Fractional polynomials were used to model non-linear risk relations with continuous variables using data from patients with recorded values to derive the fractional polynomial terms. We fitted full models initially. For consistency, we included variables from existing QRISK2 models and then retained additional variables if they had an adjusted hazard ratio of less than 0.90 or greater than 1.10 (for binary variables) and were statistically significant at the 0.01 level.”^136^

### Item 8b: specify the type of model, all model building procedures (eg, any predictor selection and penalisation), and method for validation

As for any manuscript, enough detail should be reported to allow a knowledgeable reader with access to the original data to verify the reported results. Readers should also be able to understand the reasons for the approaches taken. The key features to consider and report for a prediction model development study are the type of model fitted, predictor selection, interaction effects, strategies to avoid overfitting, and internal validation (following the original TRIPOD item 10b).^1^

Developing a prediction model can be highly data driven, which can contribute towards overfitting and optimism. As discussed in the original TRIPOD E&E,^2^ selection of predictors based on the strength of unadjusted (univariable) associations should be avoided.^137^ Potentially relevant predictors might be rejected owing to nuances in the dataset or confounding by other predictors. For prediction model studies using clustered datasets, a very large number of candidate predictors could be available, and important predictors might differ across clusters. Unfortunately, guidance to help resolve these issues is currently lacking. In all cases, researchers should explain how they selected candidate predictors or cite relevant background material. When possible, researchers should use previous knowledge or a systematic literature review to help with the selection of candidate predictors.

Researchers should also explain how they selected the candidate predictors to be included in the final model. Although including all candidate predictors can make sense, methods such as stepwise elimination are often used. However, penalised methods such as LASSO and elastic net are increasingly preferred to minimise the risk of overfitting.^138^ ^139^ Regardless of the modelling approach, researchers should consider stability investigations to assess how robust the developed model is to small perturbations of the data.^137^ Several penalised estimation procedures have been proposed for clustered data.^140^ ^141^ ^142^ Researchers can also shrink the prediction model's regression coefficients after estimation, determining the shrinkage factor with a heuristic formula 143 144 145 or bootstrapping.^143^ ^145^ ^146^

All models should be internally validated to examine whether overfitting is likely to be present and to obtain realistic model performance estimates that have been corrected for optimism. Several validation techniques have been proposed. Resampling techniques such as bootstrapping can be applied to the entire IPD set or to each cluster^147^ to obtain optimism corrected estimates of model performance. Internal-external cross validation can be used to investigate the mode’s consistency and transportability across different settings and populations (see item 5 for details).^7^ ^40^ ^96^ ^97^ ^148^

Authors should fully explain any internal validation procedures and clarify whether and how heterogeneity was taken into account (see also item 8f). As detailed in the original TRIPOD E&E,^2^ all aspects of model building should be incorporated when performing internal validation using resampling methods, including predictor selection, penalisation, transformations, and interaction tests. If only the final model is used in bootstrapping or (internal-external) cross validation, important aspects of model uncertainty are ignored, leading to overoptimistic internal validation results.

#### Example of individual participant data meta-analysis

“We used a backwards elimination procedure with bootstrapping to select predictors of the composite survival outcome. Predictors that were selected in more than 70% of the bootstrap resamples entered a multivariable prediction model. Data from the 14 cohorts were combined using the internal-external cross-validation framework. This developed a model for predicting our composite survival endpoint in all but one cohort, after which its external validity was evaluated in the omitted cohort. The process was repeated for all 14 cohorts (every cohort being omitted once). For model development, we used multivariable Royston-Parmar survival models rather than Cox survival models, to facilitate the calculation of absolute risks in individual patients when implementing the model in clinical practice. We assumed a common baseline hazard for all cohorts, but also reported values of cohort-specific baseline hazard functions, which might help to tailor predictions to different populations. We used fractional polynomials to identify non-linear relationships with our composite survival endpoint.”^74^

#### Example using electronic health records data

“For derivation of the risk prediction model, we initially included all candidate predictors in a multivariable logistic regression model. We fitted a clustering term to take account of consecutive pregnancies within women during the study period and used fractional polynomials to model potential non-linear relations between outcome and continuous predictors. Through backwards elimination, we excluded (except for age at delivery, which was considered a prior predictor and retained in the model regardless of statistical significance) candidate predictors from the multivariable model that were not statistically significant (P>0.1 based on change in log likelihood). After elimination, we reinserted excluded predictors into the final model to further check whether they became statistically significant. We also rechecked fractional polynomial terms at this stage and re-estimated them if necessary . . . We then did internal validation to correct measures of predictive performance for optimism (over-fitting) by bootstrapping 100 samples of the derivation data. We repeated the model development process in each bootstrap sample (as outlined above, including variable selection) to produce a model, applied the model to the same bootstrap sample to quantify apparent performance, and applied the model to the original dataset to test model performance (calibration slope and C statistic) and optimism (difference in test performance and apparent performance). We then estimated the overall optimism across all models (for example, derive shrinkage coefficient as the average calibration slope from each of the bootstrap samples). To account for over-fitting during the development process, we multiplied the original β coefficients by the uniform shrinkage factor in the final model. At this point, we re-estimated the intercept on the basis of the shrunken β coefficients to ensure that overall calibration was maintained, producing a final model.”^116^

### Item 8c: describe how any heterogeneity across clusters (eg, studies or settings) in model parameter values was handled

Prediction model research that uses a clustered dataset must consider if and how to handle potential heterogeneity in the prediction model’s parameter values.^7^ ^14^ ^38^ ^40^ Such heterogeneity commonly occurs because of the spectrum effect, when the distribution of participant characteristics, including the outcome, varies across different settings and populations.^56^ ^57^

For example, the prognostic effect of a cancer biomarker might vary or interact with other predictors, such as the stage of disease or treatment received. It might therefore have a non-linear relation with the outcome risk. However, such interactions and non-linear trends are often missed or mis-specified during model development. Biomarkers are also often measured differently (eg, using equipment from different manufacturers or a different assay or technique), recorded at different times (eg, before or after surgery), or quantified differently (eg, using a different cut-off point to define high and low values) across settings. Clusters can differ in many other ways, including treatment strategies, disease and outcome definitions, and follow-up lengths. All of these problems can lead to heterogeneity in predictor effects.^149^ ^150^

Even more common is heterogeneity in the baseline risk, model intercept, or baseline hazard rate. Here, the average prevalence or incidence rate of the predicted outcome varies across the included clusters.^37^ This heterogeneity is caused, for example, by different standards of care, treatment strategies, or treatment start points, such as earlier diagnosis and treatment of diseases in some populations through a screening programme.^8^ The intercept or baseline hazard rate of a developed prediction model might therefore not be transportable from one population to another, leading to predicted risks that are systematically too low or too high.

Prediction model development studies of participants from multiple sources should report if and how heterogeneity in the model parameters was identified and dealt with.^7^ ^40^ For example, a logistic regression model can be fitted incorporating random effects that allow for heterogeneity in the intercept and predictor effects. Alternatively, a separate, fixed effects intercept can be estimated per study, to allow for study specific, baseline risk estimates.^7^ For survival data, the baseline hazard can be estimated by adopting flexible parametric methods during model development.^151^ For example, the Royston-Parmar model estimates the baseline hazard using restricted cubic splines.^152^ An overview of modelling approaches that can be adopted in large clustered datasets is given in table 3.

**Table 3:** Possible approaches to account for the presence of clustering and heterogeneity

Model parameter and modelling choices to account for clustering	Modelled difference between studies	Modelling approaches
**Intercept term**		
Common effect	All clusters share a common intercept term	GLM
Random effects	Clusters might have a different intercept term. The intercept terms are assumed to be related, and assumed to follow a certain (usually normal) distribution across clusters.	GLMM^153^ ^154^
Fixed effects (stratification)	Clusters might have a different intercept term. The intercept terms are unrelated, and estimated separately for each cluster. Different groups of clusters might also have a different intercept term. Clusters could be stratified by a categorical (eg, secondary versus tertiary care) or continuous (eg, inpatient capacity) variable.	GLM including the cluster membership or the cluster level variable as an additional covariate^154^
**Baseline hazard function**		
Common	All clusters share a common baseline hazard function	Survival model (eg, CPH model, RP model)
Random effects	All clusters share a common shape of the baseline hazard function, but its magnitude can vary across clusters. Briefly, the baseline hazard functions are assumed to be proportional across clusters, and their magnitude are assumed to follow a certain distribution.	Frailty models for survival data^151^ ^155^-^157^ (eg, hierarchical CPH model)
Fixed effects	All clusters share a common shape of the baseline hazard function, but its magnitude might vary across clusters. Briefly, the baseline hazard functions are assumed to be proportional across clusters. The magnitude of the baseline hazard function is then estimated separately for each cluster.	Survival model where cluster membership is included as an additional covariate^151^
Stratification	All clusters might have a different baseline hazard function, which is estimated separately for each cluster. No relation exists between the baseline hazard function of different clusters.	Survival model where cluster membership is included as stratum variable^151^
**Regression coefficient**		
Common effect	The magnitude of the regression coefficient is identical for all clusters.	Any regression or survival model
Random effects	The magnitude of the regression coefficient might vary across clusters. The regression coefficients are assumed to be related, and assumed to follow a certain (usually normal) distribution.	GLMM,^153^ ^154^ frailty model for survival data
Fixed effects (Stratification)	The magnitude of the regression coefficient might vary across clusters. The regression coefficients are unrelated, and estimated separately for each cluster.	Regression or survival model with an interaction term between the predictor and cluster membership
**Variance of the residual error**		
Common	The variance of the residual error is common across all clusters	GLM
Random effects	The variance of the residual error might vary across clusters. The error variances are assumed to be related, and assumed to follow a certain (usually inverse gamma) distribution across clusters.	GLMM^154^
Fixed effects (stratification)	The residual variance is estimated separately for each cluster, and is unrelated across clusters.	GLM with heteroscedasticity

GLM=generalised linear regression model; GLMM=generalised linear mixed regression model; CPH=Cox proportional hazards; RP=Royston-Parmar. More specific details about the implementation of each approach are available from the literature, which generally focus on binary outcomes,^154^ ^158^ continuous outcomes,^154^ time-to-event outcomes,^151^ ^154^ ^159^ and other outcomes.^154^

In general, researchers should limit any heterogeneity in baseline risk and predictor effects while keeping the model’s overall performance sufficiently high.^40^ Although several methods for this purpose have been proposed,^64^ ^160^ formal guidance is currently lacking. Finally, users can also update the model’s parameters at implementation to recalibrate its predictive performance to the new population.^11^ Authors should then report the updating strategy (see also item 8g).

#### Example of individual participant data meta-analysis

“Predictor effects might differ across the low and high prevalence settings, and we tested these differences by using interaction terms between setting and all other variables. We also tested interactions between symptoms and sex, symptoms and age, and symptoms and diabetes. Linear effects of age and the log transformed coronary calcium score were tested by including a restricted cubic spline function with three knots.”^161^

#### Example using electronic health records data

“A random-intercept hierarchical logistic regression model was employed to adjust for clustering at hospital level . . . Both fixed and random effects models were tested without substantial difference, and the results of the more robust latter method are reported here.”^162^

### Item 8d: for validation, describe how the predictions were calculated

Authors should clearly describe how they calculated risk predictions based on an existing model for individuals during validation. Problems can arise when, for example, the original model development paper only reports some of the model’s parameters (eg, regression coefficients) or when some of the model’s predictors are not available in the validation dataset. Authors should clarify which estimates they used, particularly when more than one would be valid. For instance, researchers validating a prediction model with random intercept terms can choose to use a study specific intercept term,^7^ use the pooled intercept term,^7^ or integrate over the distribution of random intercept terms.^163^ These approaches typically lead to different calibration performance.^55^

As discussed in the TRIPOD E&E,^2^ prediction models are often presented in different formats, such as formulas, nomograms, or web calculators.^164^ Authors should clearly explain which format was used to obtain predictions. When IPD are available from multiple clusters, a common problem is systematically missing predictors, where some predictors are completely unavailable in a subset of clusters. Authors should then report how predictions were made in this subset (item 7d). Authors should also clearly state if it was not possible to validate a certain model.

#### Example of individual participant data meta-analysis

“The final models were presented in a score chart, with scores based on the regression coefficients in the proportional odds models. Coefficients were scaled such that the same rounded score was obtained for predictors that were used across the different models (e.g., age, motor score, pupils). Logistic regression was subsequently used to calibrate the risks of mortality and unfavourable outcome according to the scores, with the model intercept referring to the Tirilazad international trial. This intercept was chosen since it represented typical proportions of mortality and unfavourable outcome.”^165^

#### Example using electronic health records data

“The original model was developed to predict 10-year patient survival from 90 days after the start of renal replacement therapy. It was based on age at the start of RRT [renal replacement therapy], primary renal disease, sex and therapy at 90 days. The formula for the survival probability at time *t*, S(*t*), is S(*t*)=exp(−H(*t*)). Here, H(*t*) is the cumulative hazard that is calculated from the baseline hazard (H_0_) as H(*t*)=H_0_(*t*)*exp(prognostic index). The prognostic index can be calculated using the values of the four predictors for a specific patient (Table 1) together with their parameters estimates.”^69^

### Item 8e: specify all measures used to assess model performance (eg, calibration, discrimination, and decision curve analysis) and, if relevant, to compare multiple models

As stated in the original TRIPOD guidance, all prediction modelling papers should characterise the model’s predictive performance with (at least) an assessment of calibration and discrimination.^1^ ^2^ ^166^ Calibration assesses the accuracy of the estimated risks and can be evaluated at different levels using various measures.^95^ ^166^ ^167^ For example, the calibration intercept is an evaluation of calibration-in-the-large. This property can also be assessed by the ratio of the observed number of events over the expected number of events. For internal validation, an assessment of calibration slope might suffice, although the calibration intercept and a calibration plot can be provided as well. For external validation, calibration plots are strongly preferred, accompanied by the calibration intercept and slope.

Discrimination refers to a prediction model’s ability to differentiate between individuals who do and do not experience the outcome event. The most general and widely reported discrimination measure is the c statistic (c index), which can be used for both logistic and survival models. The c statistic is the area under the receiver operating characteristic curve for logistic prediction models.^168^ Because different versions of the c statistic exist, authors should clearly state which version they calculated. Recently, several extensions of the c statistic have been proposed for use in clustered data.^169^ ^170^ Discrimination in survival models can also be assessed using Royston’s D statistic.^171^

Authors can also report other overall measures of their model’s predictive performance, including the Brier score and generalised R^2^ values, such as the Cox-Snell R^2^ and Nagelkerke R^2^. In addition, it is often highly useful to report measures of clinical usefulness,^166^ for example, based on net benefit and decision curve analysis.^172^ ^173^ These measures offer insight into the consequences of making clinical decisions using a prediction model at a specific threshold (eg, to treat if the predicted risk is ≥10%).

Model performance measures can also be used to compare models. For example, the difference in the c statistic can be calculated to compare the discrimination performance of two competing models in the same data. Models’ clinical usefulness can be compared by calculating the difference in the net benefit or decision curve for two competing models, and against blanket treatment strategies to treat all or to treat none. The test trade-off can be calculated to quantify whether one strategy’s increase in net benefit is worth its additional cost, versus using the next best strategy. The test trade-off can be expressed and reported in terms of true positives and true negatives.^173^

#### Example of individual participant data meta-analysis

“The predictive performance of each model was examined using measures of discrimination and calibration, firstly in the IPD for each available dataset and then across datasets at the meta-analysis level . . . We considered [c statistic] values over 0.7 to be most promising, given previously reported values in the literature, while noting the width of confidence intervals . . . Calibration was assessed using two measures: calibration slope, which is the slope of the regression line fitted to the relationship between predicted and observed risk probabilities on the logit scale (ideal value of 1); and calibration-in-the-large, which indicates whether risk predictions are systematically too high or too low (ideal value of 0). We produced calibration plots in each dataset to visually compare observed and predicted probabilities when there were enough events to categorize participants into risk groups. The predicted probability of pre-eclampsia for each individual was obtained by pooling the imputation-specific estimates of the model's linear predictor and then applying the logit transformation. For each pre-eclampsia outcome (early, late or any onset), we compared prediction models using decision curve analysis in the datasets used most frequently in the external validation of the prediction models, enabling within-dataset comparison of the models. Decision curves show the net benefit (i.e. benefit versus harm) over a range of threshold probabilities (i.e. for treating women with a predicted risk above the threshold value) and can be compared to the treat all and treat none strategies.”^115^

#### Example using electronic health records data

“As in previous studies, we calculated the *D* statistic (a measure of discrimination where higher values indicate better discrimination), R^2^ value (explained variation where higher values indicate a greater proportion of variation explained by the model in time to diagnosis of type 2 diabetes) based on Royston’s D statistic, and Harrell’s c-statistic at 10 years and combined these across datasets using Rubin’s rules. Harrell’s c-statistic is a measure of discrimination similar to the receiver operating characteristic statistic but takes account of the censored nature of the data. Calibration was assessed by comparing the mean predicted risks at 10 years with the observed risk by 10th of predicted risk. The observed risks were obtained using the Kaplan-Meier estimates evaluated at 10 years. We also evaluated performance by subgroups for each age band (<40, 40 – 59, ≥60 years), ethnic minority group, and comorbidity and treatment group. We calculated calibration slopes. Performance was also evaluated by calculating Harrell’s c-statistics in individual general practices and combining the results using meta-analytical techniques.”^136^

### Item 8f: describe how any heterogeneity across clusters (eg, studies or settings) in model performance was handled and quantified

Prediction model performance often varies across settings and populations,^7^ ^14^ ^26^ which could be due to differences in case mix variation. This case mix variation is similar to the spectrum effect (as described in item 8c).^56^ ^57^ Discrimination performance can deteriorate when a model is used in populations with a more homogeneous case mix than the development population, or improve when the case mix is more heterogeneous.^15^ ^26^ For example, the Wells score was developed for outpatients in secondary care to predict deep vein thrombosis. When applied to a substantially different population (patients in hospital), its discrimination performance was only slightly better than relying on chance alone.^174^

Prediction model performance can also vary because of the use of invalid model parameters (eg, regression coefficients). Differences in how predictors are measured across data sources,^46^ ^104^ disease and outcome definitions, treatment strategies, and measurement error 60 105 106 can all affect the validity of estimated model parameters across clusters.

Researchers are strongly encouraged to assess heterogeneity in model performance across populations, settings, and time,^40^ ^46^ ^62^ ^97^ and to report how they accounted for this heterogeneity. For instance, the presence of heterogeneity in clustered data can be explored by validating the prediction model separately in each study and depicting the resulting performance estimates in a forest plot. A random effects meta-analysis can then be conducted to summarise the model’s average performance, quantify the extent of heterogeneity between studies, and calculate the model’s likely performance in new settings by constructing a prediction interval.^46^ ^175^ ^176^ ^177^ ^178^ Authors should report statistical models, estimation methods (eg, restricted maximum likelihood estimation), and methods for calculating confidence and prediction intervals, whether precise or approximate. If prediction model performance varies substantially across settings or populations, authors are recommended to investigate and describe whether this occurs because of case mix variation or invalid model parameters.^7^ ^26^

#### Example of individual participant data meta-analysis

“We summarised the performance measures across datasets using a random effects meta-analysis estimated using restricted maximum likelihood (for each performance measure separately). Summary (average) performance statistics were reported with 95% confidence intervals (derived using the Hartung-Knapp-Sidik-Jonkman variance correction). We also reported the estimate of between-study heterogeneity (τ^2^) and the proportion of variability due to between-study heterogeneity (I^2^). We used forest plots to show a model’s performance in multiple datasets, and to compare the average performance (across datasets) of multiple models.”^115^

#### Example using electronic health records data

“We also used an internal-external cross-validation (IECV) approach to evaluate the two derived prediction models over 13 geographical regions in the UK . . . Performance statistics were also summarised across regions using a random-effects meta-analysis, reporting the average performance statistic with 95% confidence interval (derived using the Hartung-Knapp-Sidik-Jonkman variance correction) and 95% prediction interval.”^66^

### Item 8g: describe any model updating (eg, recalibration) arising from the validation, either overall or for particular populations or settings

A prediction model’s performance often deteriorates when it is applied to new individuals. Researchers might then decide to tailor an existing model to specific settings or populations, for example, by recalibrating its intercept term or adjusting the overall slope by scaling all regression coefficients by a common factor. The original TRIPOD E&E states that an existing model should not be updated in new data until the model’s predictive performance in the new data has first been quantified.^2^

Simple model updates, such as intercept recalibration or overall slope adjustment, are difficult when predictor effects differ between the development and validation samples and calibration plots show inconsistent predictions across the range of predicted probabilities. Researchers might then have to re-estimate individual predictor effects.^15^ Existing methods for updating models vary in extensiveness, depending on the number of parameters to be re-estimated.^179^ Several methods have been proposed to evaluate the extent of updating needed. For instance, Snell et al proposed applying various implementation strategies (eg, recalibration) and jointly synthesising the resulting models’ calibration and discrimination performance.^177^ This method shows which modelling strategy is most likely to yield good performance in new populations. Also, closed testing procedures have been proposed for selecting an appropriate update method that balances between the amount of evidence available for updating in the new patient sample and the danger of overfitting.^180^ ^181^

Authors should clearly report which strategies for model updating they used and why, including whether they used different strategies for different data sources or clusters.

#### Example of individual participant data meta-analysis

“We recalibrated the model by comparing the average regression slope of the clinical model with the average regression slope in the validation data. A second linear predictor variable was added to the model (while maintaining both the previous linear predictor as offset variable and the new intercept) and its coefficient, the β_miscalibration_ was estimated. This coefficient reflects the miscalibration of the predictor effects in the clinical model when compared to the predictor effects in the validation data. We tested whether β_miscalibration_=0, corresponding to the hypothesis that the prediction of the clinical model (adjusted for calibration-in-the-large) fits the data well. If significant, we conclude that the overall effects of all predictors together are different in the validation data and that the model should be revised). Finally, we re-estimated the predictor effects in a validation model including the linear predictor of the clinical model as offset and the new intercept. The coefficients from this analysis refer to the difference between the re-estimated (validation coefficients) and the coefficients from the clinical model. We tested whether these differences were significantly different from zero. From these analyses we can judge which predictor effects are different in the validation data as compared to the clinical model which was based on the total cohort.”^161^

#### Example using electronic health records data

“In case the calibration plots in the validation data indicate systematic over- or underestimation of risk, we plan to explore the effect of recalibration by updating the baseline hazard for the entire validation dataset or a subgroup. The baseline hazard will be updated by fitting the original model to the validation data with the linear predictor as an offset term. The updated model will then be used to estimate the updated baseline hazard at 2 and/or 5 and/or 10 years, and the calibration performance will be evaluated comparing an updated calibration plot with the original calibration plot.”^99^

### Item 9: describe any planned subgroup or sensitivity analysis (eg, assessing performance according to sources of bias, participant characteristics, setting)

Authors can perform additional analyses to test the robustness of a prediction model’s performance. Sensitivity analyses are used to examine whether a model’s overall predictive performance is affected by, for example, changes in key modelling assumptions or participant eligibility criteria. Subgroup analyses are used to study performance in subgroups defined by important characteristics at the patient level or cluster level.

In studies using multiple data sources, variation according to differences in design or conduct between data sources might be of particular interest. Access to IPD from multiple data sources allows researchers to examine whether a model’s performance is comparable across settings or studies with different design characteristics or participant subgroups.

Shortcomings in the design and conduct of prediction model studies can lead to biased results. Researchers can also perform a sensitivity analysis using only studies of good quality and report how performance measures change.

Sometimes researchers develop and evaluate a simplified prediction model alongside their full model. For instance, the regression coefficients of a developed prediction model are sometimes assigned integer scores, creating a point score system.^164^ A table or figure is then provided to translate the total sum score into an absolute risk prediction. This strategy sometimes involves categorising individuals into a risk group (eg, low, intermediate, and high risk). As stated in the original TRIPOD guidance,^1^ authors should explain how the risk groups were defined (eg, boundaries used) and chosen. If the risk groups are to be used for clinical decision making, authors should explain how they chose the number of groups and group boundaries. Thresholds that are derived using statistical criteria (eg, Youden index) are usually not clinically relevant and should be avoided.^182^ Authors should report the performance of the full model and of simplified models, because the categorisation and simplification used in their construction often leads to a loss of information and therefore worsened predictive performance.

#### Example of individual participant data meta-analysis

“Performance of each prediction model will also be summarised according to the risk of bias (using PROBAST [prediction model risk of bias assessment tool]) where there are enough studies to do so; for example, summarising model performance statistics for only the studies that are low risk of bias for specified criteria to assess whether there is less heterogeneity in performance.”^132^

#### Example using electronic health records data

“Because age is a strong predictor for survival, we performed an additional sensitivity analysis, stratifying calibration and discrimination analyses by age. For comparison of model performance, we also stratified by sex. Further, as some countries only had good data completeness in more recent years, we stratified the calibration and discrimination analysis by the starting year of renal replacement therapy.”^69^

## TRIPOD-Cluster checklist: results

### Item 10a: describe the number of clusters and participants from data identified through to data analysed

Readers need to understand the original sources of the participants included in the analysis to judge the context in which a prediction model was developed or validated, and thus can be generalised to. Authors should therefore describe how they selected their data sources from all possible data sources and, if applicable, how they separated the data into development and validation sets. The flow of participants in a study with clustered data can be given in the text, a table, or a flow diagram. Flow diagrams are recommended because they visually clarify how the study samples were established for development and validation. This information should be reported for each cluster, unless the study used a prohibitively large number of clusters.

A flow diagram for a prediction model study based on clustered data should start with the original sources of the analysed participants. Diagram steps can show the eligibility criteria and data availability. Authors can also include information such as the numbers of participants with missing observations, numbers of outcome events, and number of clusters.

#### Example of individual participant data meta-analysis

 See figure 3.

**Fig 3:**
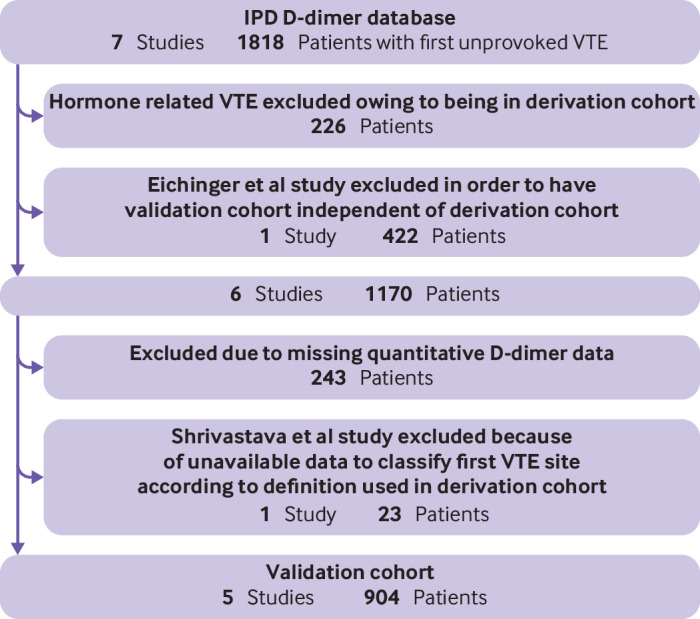
Example flow chart of an individual participant data (IPD) meta-analysis, showing which data were eligible and used for external validation. VTE=venous thromboembolism. Adapted from reference 183^183^ with permission from Wiley, copyright 2015 International Society on Thrombosis and Haemostasis

#### Example using electronic health records data

See figure 1 from reference 184, showing a patient flowchart of which data were used for development and validation of the PreOperative Score to predict PostOperative Mortality (POSPOM).^184^

### Item 10b: report the characteristics overall and, where applicable, the characteristics for each data source or setting (including the key dates, predictors, treatments received, sample size, number of outcome events, follow-up time, and amount of missing data)

Authors should report the details of each data source used for developing the prediction model, such as key dates, setting, participant demographics, sample size, predictors, received treatments, outcome, and amount of missing data. Readers can then assess the target population and deduce relevant information, such as available tests, treatments, and state-of-the-art medical technology during data collection. The main characteristics can be shown in a table, including medians or means and ranges of continuous predictors, outcome details, and the number of missing observations for each predictor or outcome. Authors should clearly report the ranges of all continuous predictors, particularly predictors in the model, to indicate to whom the model might apply.

Where possible, authors should include a summary of the main characteristics of each cluster (table 4). Readers need this information as differences in these characteristics can lead to heterogeneity in the model’s parameter values and performance. However, if the study used a prohibitively large number of clusters, authors could instead include a summary of all clusters (table 5) or summarise groups of clusters (eg, summary by country). The case mix similarity between clusters can also be summarised using a membership model that quantifies to which extent patients from different clusters can be separated.^15^ ^26^

**Table 4:** Example of individual participant data meta-analysis, comparing distribution of important variables against development data, in studies developing or validating a prediction model in clustered data. Adapted from: Apolo AB, Ostrovnaya I, Halabi S, et al. Prognostic model for predicting survival of patients with metastatic urothelial cancer treated with cisplatin-based chemotherapy. *J Natl Cancer Inst* 2013;105:499-503 by permission of Oxford University Press^185^

Characteristic	Development			Validation (cohort 4)
	Cohort 1	Cohort 2	Cohort 3	
Total No of participants	203	45	60	74
Total No of deaths	184	37	53	68
Median (95% CI) survival (months)	14.8 (12.1 to 16.7)	18 (12.0 to 29.7)	16.4 (14.6 to 22.9)	12.7
Sex (%)				
Male	80	73	77	78
Female	20	27	23	22
Median age (years)	63	63	62	64
Median KPS (%)	80	80	90	90
Visceral disease (%)	49	40	33	69
Bone	26	11	10	18
Liver	13	13	10	31
Lung	26	22	18	43
No of risk factors (%)				
0	33	55	62	30
1	45	41	35	65
2	22	4	3	5

Baseline characteristics of an individual participant data meta-analysis to develop and validate a nomogram for overall survival in metastatic urothelial cancer patients treated with cisplatin-based chemotherapy. KPS=Karnofsky performance status; CI=confidence interval.

**Table 5:** Example of electronic health records data comparing distribution of important variables against development data, in studies developing or validating a prediction model in clustered data. Adapted from: Collins GS, Altman DG. Identifying patients with undetected renal tract cancer in primary care: an independent and external validation of QCancer® (Renal) prediction model. *Cancer Epidemiol* 2013;37:115-20, with permission from Elsevier^186^

Risk predictor	QRESEARCH		THIN (external validation; n=2 145 133)
	Development (n=2 359 168)	Internal validation (n=1 240 722)	
Median age (years)	50.1 (SD 15.0)	50.1 (SD 14.9)	48 (IQR 38-61)
Smoking status (No (%))			
Non-smoker	1 197 521 (50.8)	626 066 (50.5)	860 217 (40.1)
Ex-smoker	425 611 (18.0)	228 649 (18.4)	311 924 (14.5)
Current smoker amount not recorded	71 603 (3.0)	39 396 (3.2)	282 534 13.2)
Light smoker (<10 cigarettes/day)	148 703 (6.3)	80 103 (6.5)	133 657 (6.2)
Moderate smoker (10-19 cigarettes/day)	180 509 (7.7)	96 175 (7.8)	203 954 (9.5)
Heavy smoker (≥20 cigarettes/day)	134 688 (5.7)	73 981 (6.0)	183 590 (8.6)
Smoking status not recorded	200 533 (8.5)	96 352 (7.8)	169 257 (7.9)
Current symptoms and symptoms in the preceding year (No (%))			
Haemoglobin <11 g/dL recorded in past year	29 720 (1.3)	16 169 (1.3)	16 961 (0.8)
Abdominal pain	230 584 (9.8)	128 721 (10.4)	253 344 (11.8)
Appetite loss	10 287 (0.4)	5531 (0.4)	6097 (0.30)
Weight loss	25 897 (1.1)	14 464 (1.2)	29 369 (1.4)
Haematuria	43 850 (1.9)	25 553 (2.1)	37 810 (1.8)
Previous diagnosis of cancer apart from renal tract cancer at study entry	51 119 (2.2)	27 163 (2.2)	49 303 (2.3)

Baseline characteristics of the development and an external validation cohort of QCancer, which predicts the risk of having undiagnosed lung, ovarian, colorectal, gastro-oesophageal, renal, or pancreatic cancer. SD=standard deviation; IQR=interquartile range; THIN=Health Improvement Network.

#### Example of individual participant data meta-analysis

“This study was based on 26 cohort studies of the COPD [chronic obstructive pulmonary disease] Cohorts Collaborative International Assessment (3CIA) consortium. Details have been reported elsewhere (and summarised in Table 2) . . . The cohorts varied greatly in terms of geographic location, sample size, and number of events and included a broad spectrum of patients with COPD from primary, secondary, and tertiary care settings (Table 2).”^68^

#### Example using electronic health records data

“After three participants were excluded from the analysis as their death date was recorded before their start date (likely due to clerical error), there were a total of 502 625 participants in the entire cohort who were followed up for a total of 3 508 454 person-years, resulting in 14 418 deaths (2.9%). Table 1 describes in detail the baseline characteristics of the study population.”^187^

### Item 10c: for validation, show a comparison with the development data of the distribution of important variables (demographics, predictors, and outcome)

Authors reporting a validation should include the details of each data source and compare them with the original development data (see also item 7c).^15^ The main characteristics can be shown in a table, including medians or means and ranges of continuous predictors, outcome details, and the number of missing observations for each predictor or outcome. Where possible, a summary of the main characteristics should also be reported for each cluster, because differences in these characteristics could explain heterogeneity in prediction model performance. However, if the study used a prohibitively large number of clusters, authors could include a summary across all clusters (or stratified by a cluster level variable (or both)), including the range of values across clusters (eg, ranges of mean age, proportion of male participants, and number of participants and events).

#### Examples

See table 4 and table 5.

### Item 11: report results of the risk-of-bias assessment in individual clusters

Regardless of which critical appraisal or risk-of-bias assessment tool was used (see item 7b), readers need access to the appraisal results to understand the model’s risk of bias and applicability. We encourage authors to include at least a narrative summary of the quality appraisal, as is also recommended by item 22 of the PRISMA-IPD statement.^188^ A table (eg, table 6) or graphical presentation of the assessments per cluster is preferred where possible. However, this summary alone is not sufficient. It should be followed by, for example, a sensitivity analysis guided by the risk-of-bias assessments (see item 15) and a discussion of how the observed patterns in risk of bias affect interpretation of the pooled results and inferences (see item 16c).

**Table 6:** Example of an individual participant data meta-analysis reporting result of the risk-of-bias assessment for the included studies. Information adapted from information licensed under the Non-Commercial Government Licence version 2.0^115^

Study/dataset	Domain: predictors					
	Predictors defined in a similar way for participants	Predictors defined in a similar way to model development study	Predictors assessed without knowledge of outcome data	All predictors available at the time model is to be used	Risk	Rationale
Allen	Yes	NA	Yes	Yes	Low	Responses all yes to signalling questions
ALSPAC	Yes	NA	Yes	Yes	Low	Responses all yes to signalling questions
Chappell	Probably no	NA	Probably yes	Yes	High	Historical data and definition of predictor may differ over time
EMPOWAR	Yes	NA	Yes	Yes	Low	Responses all yes to signalling questions
Poston 2006	Yes	NA	Yes	Yes	Low	Responses all yes to signalling questions
Poston 2005	Yes	NA	Yes	Yes	Low	Responses all yes to signalling questions
UK SCOPE	Yes	Yes	Yes	Yes	Low	Responses all yes to signalling questions
St Georges	Probably yes	NA	Probably yes	Yes	Low	Responses all yes to signalling questions
Velauthar	No information	NA	No information	Yes	Unclear	No information to make assessment

Risk-of-bias assessment for predictors available in an individual participant data meta-analysis to develop and validate predictions models for pre-eclampsia. NA=not applicable; SCOPE=Screening for Pregnancy Endpoints; ALSPAC=Avon Longitudinal Study of Children and Parents; EMPOWAR=Efficacy of Metformin in Pregnant Obese Women (a randomised controlled trial).

#### Example of individual participant data meta-analysis

“Quality assessment using the PROBAST tool resulted in 40% (4/10) of the included IPD datasets being classified as low risk of bias, while 10% (1/10) were classified as high risk of bias and 50% (5/10) were classified as unclear risk of bias. All of the included datasets had a low risk of bias in the domain of participant selection. For the domain of predictors, eight (80%) had a low risk of bias, while one each had a high and an unclear risk of bias assessment [table 6] . . . The risk of bias in the outcome domain was unclear for six of the included datasets and low risk in four.”^115^

### Item 12a: report the results of any heterogeneity assessments across clusters that led to subsequent actions during model development (eg, inclusion or exclusion of particular predictors or clusters)

Authors should report the findings of any investigations of heterogeneity during model development (see item 8c), because these findings could lead to important decisions about model specifications and including or excluding individuals or clusters. For example, in an IPD-MA, Ensor et al investigated heterogeneity in the baseline hazard rate by estimating a Royston-Parmar proportional hazards model separately in each study.^98^ They found a similar shape but with different magnitudes across studies. Subsequently, the developed model used a frailty term to allow for proportional baseline hazards across studies (see also table 3). The presence of heterogeneity as such, which is expected in clustered datasets, is not a sufficient reason to exclude individuals or clusters. Any exclusion of individuals or clusters should be clearly justified.

#### Example of individual participant data meta-analysis

**Fig 4:**
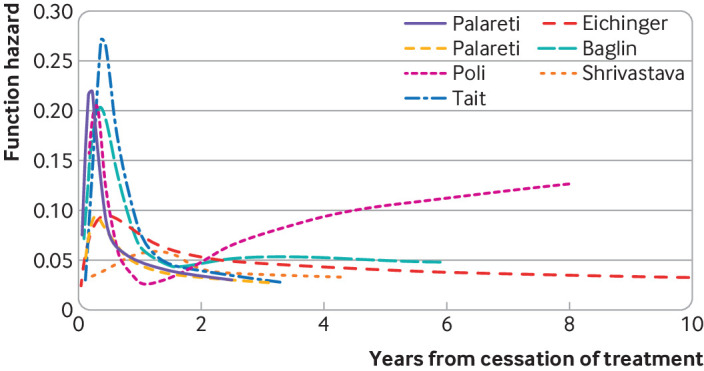
Example of an investigation of the baseline hazard function within each trial of an individual participant data meta-analysis.^98^ Figure shows the hazard of developing recurrent venous thromboembolism in each trial as a function of the number of years from cessation of treatment after a first unprovoked venous thromboembolism event. All trials had a similar peak in hazard at just under one year from cessation of treatment; however, this peak had varying magnitudes across the seven trials. Figure adapted from Ensor J, Riley RD, Jowett S, et al. Prediction of risk of recurrence of venous thromboembolism following treatment for a first unprovoked venous thromboembolism: systematic review, prognostic model and clinical decision rule, and economic evaluation. *Health Technol Assess* 2016;20:i-xxxiii, 1-190

“[Figure 4] illustrates the baseline hazard function within each trial population plotted against years from cessation of therapy. It is clear that for all trials there is a similar peak in hazard at just under 1 year from cessation of therapy; however, this peak is of varying magnitude across the seven trials. There is also a rise in the baseline hazard seen in the Poli *et al.* trial after 2 years from cessation of therapy, which is not seen in the other trials; however, this was considered to be potentially due to the small number of individuals in this trial . . . Given the differences seen in the magnitude of the hazard function for each trial, it was deemed appropriate for model development to include a random effect on the baseline hazard, to allow for variability in the baseline hazard between trials. However, given the similarities in the general shape of the baseline hazard function in individual trials, it was deemed appropriate to assume the baseline hazards for the trials were proportional to one another.”^98^

### Item 12b: present the final prediction model (ie, all regression coefficients, and model intercept or baseline estimate of the outcome at a given time point) and explain how to use it for predictions in new individuals

Prediction models should be presented in enough detail to allow predictions for individuals, either for subsequent validation studies or in clinical practice. Authors must report the explicit formula of the developed model and can choose to give a tool for generating predictions, for example, through a webpage. For instance, when using regression analysis to develop a prediction model, all estimated coefficients should be reported, including the intercept term (eg, for linear and logistic regression models) or the estimated baseline survival (eg, for survival models), either as a function of time or the values at specific time points of interest. Authors can publish the fitted model object as supplementary information (eg, a glm object in R), rather than in the main paper, if the model is too complex to report in full (eg, if spline functions are embedded). The original TRIPOD E&E describes how reported model coefficients can be used to generate a predicted probability for individuals.^1^

Prediction models based on multiple data sources sometimes include cluster specific parameters, for instance, because a separate intercept term was estimated for each cluster. Estimates for these parameter values should be reported in full, so that the model can be applied locally in each of the included clusters. If authors intend the model to be used in other countries, settings, or populations than originally studied, they should present guidance on which parameter values should be used for risk prediction. For instance, Steyerberg et al used IPD from 11 studies to develop a prognostic model with study specific intercept terms.^165^ They proposed using the intercept term of one trial for generating risk predictions in new individuals, because that trial represented the typical proportions of mortality and the unfavourable outcome in real world applications. Ueda et al used data from eight US based cohort studies to predict fatal and non-fatal cardiovascular disease worldwide.^189^ When implementing the model outside the US, they replaced the mean risk factor levels and cardiovascular disease event rates in each 5 year age group and by sex with the best current estimates of these quantities for the target country.

#### Example of individual participant data meta-analysis

“In accordance with the TRIPOD statement, all parameters and equations of our model were provided . . . The full model equation for the log (i.e. natural logarithm) cumulative odds survival function over time *t* (expressed in months) was given as follows: 

log0(t;x) = γ_0_ + γ_1_log(t) + γ_2_v_1_(log(t)) + γ_3_v_2_(log(t)) + β_1_x_1_ + β_2_β_2_ + . . . + β_8_β_x_

where:

v_j_(z) = (z−k_j_)^3^_+_ − λ_j_(z−k_min_)^3^_+_ − (1−λ_j_)(z−k_max_)^3^_+_

λ_j_ = (k_max_−k_j_) ÷ (k_max_−k_min_)

(z−a)_+_ = max (0, z−a)

“In this model, k_min_ and k_max_ represent the boundary knots of a natural cubic spline function. The 2 other internal knots, k_1_ and k_2_, were placed at the 33% and 67% quantiles of the log uncensored survival times to the composite endpoint . . . The absolute probability for remaining event-free during *t* months since ALS [amyotrophic lateral sclerosis] diagnosis is then given as (1 + exp(log0(t;x)))^−1^, where x represent the patient’s baseline covariates for the predictors x_1_, x_2_, . . . , x_8_. Estimates for k, γ, and β are reported below. Finally, cohort-specific recalibration values of γ_0_ were reported to tailor the baseline hazard function to single cohorts.”^74^

#### Example using electronic health records data

“We formed the risk equation for predicting the log odds of venous thromboembolism by using the estimated β *c*oefficients multiplied by the corresponding predictors included in our model together with the average intercept across patient clusters. This process ultimately led to an equation for the predicted absolute risk of venous thromboembolism: predicted risk=1/1 + e^−risk score^, where the “risk score” is the predicted log odds of venous thromboembolism from the developed model . . . Risk score=−9.013 + 0.94 (0.227 smoker+1.221 varicose veins+ 0.848 comorbidities (cardiac, renal, or inflammatory bowel disease)+0.721 pre-eclampsia/eclampsia+0.421 diabetes+0.502 postpartum haemorrhage+1.151 stillbirth+1.097 postpartum infection+(0.750 emergency section/0.563 elective section)+(0.165 parity of 1/0.481 parity of 2/0.566 parity of ≥3) – 0.0000798 (age at delivery)^3^+0.0000214 ((age at delivery^3^log(age at delivery))+0.00026641 BMI^3^ − 0.0000650 (BMI^3^log(BMI [body mass index])) – 22156315 (infant birth weight)^−2^+3455223.4((infant birth weight)^−2^log(baby’s birth weight)) . . . The value −9.013 is the intercept, and other numbers are the estimated regression coefficients for the predictors, which indicate their mutually adjusted relative contribution to the outcome risk. The regression coefficients represent the log odds ratio for a change of 1 unit in the corresponding predictor.”^116^

### Item 13a: report performance measures (with uncertainty intervals) for the prediction model, overall, and for each cluster

**Fig 5:**
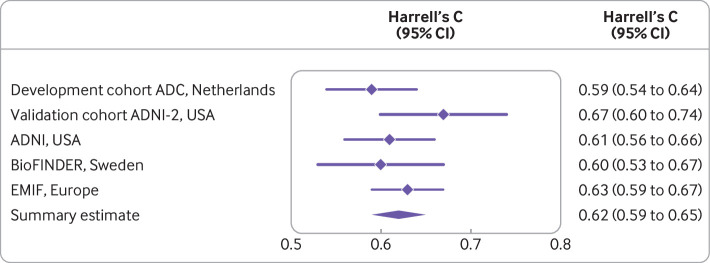
Example of an individual participant data meta-analysis reporting discrimination performance of a prediction model for clinical progression to any type of dementia in people with mild cognitive impairment, according to cohort.^190^ ADC=Amsterdam Dementia Cohort; ADNI=Alzheimer’s Disease Neuroimaging Initiative; EMIF=European Medical Information Framework for Alzheimer’s disease. Figure reproduced using data from: van Maurik IS, Vos SJ, Bos I, et al; Alzheimer’s Disease Neuroimaging Initiative. Biomarker-based prognosis for people with mild cognitive impairment (ABIDE): a modelling study. *Lancet Neurol* 2019;18:1034-44, copyright 2019 with permission from Elsevier

**Fig 6:**
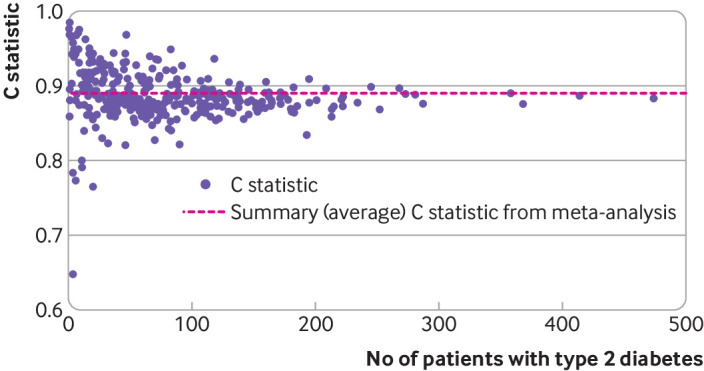
Example of electronic health records data, reporting performance measures for prediction models. Adapted from Hippisley-Cox J, Coupland C. Development and validation of QDiabetes-2018 risk prediction algorithm to estimate future risk of type 2 diabetes: cohort study. *BMJ* 2017;359:j5019^136^

All performance measures described in the methods section (item 8e) should be reported in the results section, with uncertainty (eg, confidence) intervals if applicable. If multiple models were developed or evaluated, performance measures for each model should be reported. Model development studies should report the results of internal validation or internal-external cross validation, including any optimism corrected performance measures (eg, report both the apparent and corrected c index). If a prediction model has been simplified, the performance of the original model (eg, c index of a full regression model) and the simplified model should be reported. Authors should provide estimates of model performance separately for each cluster using tables, forest plots (as depicted in fig 5), or funnel plots (as depicted in fig 6) to facilitate investigations of heterogeneity (Item 8f).

#### Examples

See figure 5 and figure 6.

### Item 13b: report results of any heterogeneity across clusters in model performance

Studies that validate a prediction model should report performance estimates for each included cluster (eg, study) in a table or figure (see item 13a). Authors should describe the corresponding results in the main manuscript and indicate whether any heterogeneity is present. Authors who performed a meta-analysis (see item 8f) should report pooled estimates and their precision alongside estimated quantities of heterogeneity (eg, variance between studies, I^2^, or a prediction interval for performance in a new population or setting).

#### Example of individual participant data meta-analysis

“Where it was possible to estimate it, heterogeneity across studies varied from small (e.g. Plasencia 2007a and Poon 2008 models had I^2^ ≤ 3%, τ^2^ ≤ 0.002) to large heterogeneity (e.g. Goetzinger 2010 and Odibo 2011a models had I^2^ ≥ 90%, τ^2^ ≥ 0.1) for the C-statistic (on the logit scale), and moderate to large heterogeneity in the calibration slope for about two thirds (8/13, 62%) of all models validated in datasets with around 100 events in total.”^115^

#### Example using electronic health records data

“The calibration slope, with the prognostic index as the only predictor, is 0.995 for the complete ERA-EDTA [European Renal Association – European Dialysis and Transplant Association] Registry cohort. For the separate countries, the slopes differ from 0.922 to 1.088.”^69^

### Item 14: report the results from any model updating (including the updated model equation and subsequent performance), overall and for each cluster

If authors tailored an existing model for specific settings or populations (see item 8g), they should fully report estimates of the predictive performance of both the updated and original models, giving at least discrimination and calibration measures with indication of heterogeneity if applicable. For example, authors should show calibration plots before and after updating. Results in tables and figures should be clearly labelled as pertaining to the original model or the updated model. If multiple updating methods were used, results should also clearly specify to which method a particular set of results correspond. If a prediction model was updated on the basis of validation study results and the updated model is recommended for future use, the authors should fully report the updated model equation following item 13a. Updates for distinct data sources, settings, or populations should be presented separately.

#### Example of individual participant data meta-analysis

“Logistic recalibration showed no significant differences between the overall hospital specific effects of the predictors compared with the overall effects of the predictors in the clinical model. When re-estimated in specific datasets, the predictor effects were not significantly different from the predictor effects in the clinical model, except for the effect of typical chest pain for Azienda Ospedaliero Universitaria Parma. The results indicated that predictor effects were similar across datasets . . . [table 7] . . . The need for an updated model was evident by our results showing that the Duke clinical score significantly overestimated the probability of coronary artery disease.”^161^

**Table 7:** Example table showing results from model validation and subsequent updating^161^

	Clinical model (n=5677)	Parma (n=1241)	Miami (n=821)	Innsbruck (n=668)	Rotterdam (n=471)
**Prediction model performance**					
C statistic (95% CI)	0.79	0.78 (0.74 to 0.81)	0.80 (0.74 to 0.85)	0.78 (0.73 to 0.82)	0.81 (0.76 to 0.88)
Calibration-in-the-large	—	0.334 (P=0.001)	−0.046 (P=0.76)	0.113 (P=0.33)	0.344 (P=0.01)
Recalibration slope	—	−0.117 (P=0.14)	0.046 (P=0.75)	0.023 (P=0.85)	0.097 (P=0.48)
**Model coefficients**					
Intercept	β= −7.539	δ=1.010 (P=0.14)	δ= −0.292 (P=0.77)	δ= −0.355 (P=0.72)	δ= −0.420 (P=0.72)
Age	β=0.062	δ= −0.003 (P=0.72)	δ= −0.015 (P=0.29)	δ= −0.018 (P=0.18)	δ= −0.013 (P=0.43)
Male sex	β=1.332	δ= −0.015 (P=0.94)	δ=0.199 (P=0.56)	δ= 0.184 (P=0.50)	δ= 0.161 (P=0.59)
Atypical chest pain	β=0.633	δ= −0.470 (P=0.06)	δ= −0.782 (P=0.05)	δ= −0.569 (P=0.17)	δ= −0.311 (P=0.53)
Typical chest pain	β=1.998	δ= −0.615 (P=0.01)	δ= −0.485 (P=0.44)	δ= −0.839 (P=0.14)	δ= −0.194 (P=0.70)
Diabetes	β=0.828	δ=0.241 (P=0.32)	δ=0.088 (P=0.79)	δ= −0.043 (P=0.88)	δ=0.038 (P=0.92)
Hypertension	β=0.338	δ= 0.096 (P=0.59)	δ= −0.433 (P=0.18)	δ= −0.274 (P=0.30)	δ=0.219 (P=0.45)
Dyslipidaemia	β=0.422	δ= −0.131 (P=0.43)	δ=0.008 (P=0.98)	δ= −0.042 (P=0.89)	δ= −0.117 (P=0.70)
Smoking	β=0.461	δ= −0.121 (P=0.57)	δ=0.449 (P=0.77)	δ=0.022 (P=0.93)	δ=0.222 (P=0.50)

β=estimated regression coefficient; δ=difference between the hospital-specific predictor effects and the predictor effects as estimated in the development data. P values above 0.05 support the validity of the developed prediction model in a specific hospital (Parma, Miami, Innsbruck, or Rotterdam).

#### Example using electronic health records data

“In our Swedish cohort, 521 women had postpartum venous thromboembolism with an absolute rate of 7.9 per 10000 deliveries. Applying our final risk prediction model to the independent population after recalibration of the intercept gave a C statistic of 0.73 (0.71 to 0.75) and excellent calibration, with the calibration slope only slightly above 1.”^116^

### Item 15: report results from any subgroup or sensitivity analysis

Authors should report the results of any subgroup or sensitivity analyses (see item 9), regardless of what the results show. One of the key advantages of doing prediction research in clustered data are that the increased sample size can allow meaningful subgroup analyses. The results should focus on the various measures of performance of interest for each subgroup and whether the model performs substantially differently between subgroups. Important differences can indicate that the model coefficients need to be tailored or adjusted for each subgroup to ensure adequate performance (eg, in terms of calibration), or that a different model is needed in particular subgroups.

#### Example of individual participant data meta-analysis

“Re-estimating the parameters did not increase model fit for the models with dementia as the outcome, both with and without centre-specific effects (table 3). For the CSF [cerebrospinal fluid] biomarkers model with Alzheimer’s disease dementia as the outcome, re-estimating the parameters did increase model fit (appendix p9). Inclusion of centre-specific effects did not improve any of the models relative to those without centre-specific effects (table 3). Notably, inclusion of centre-specific effects did not result in a difference in progression probabilities on an individual level (data not shown). Additional analyses further supported this finding, as we found that centre-specific effects were not confounded by measurement methods for MRI [magnetic resonance imaging] and CSF (appendix pp 10-12). Therefore, we favoured models without centre-specific effects to increase generalizability.”^190^

#### Example using electronic health records data

“Good agreement between observed and predicted risks was observed in each geographical region cohort for THR [total hip replacement]. For those at highest risk of TKR [total knee replacement] (>10th decile), the observed risk was slightly higher than the predicted risk from the model in Northern Ireland, Scotland and Wales. We hypothesised that this slight miscalibration could result from systematic differences between these devolved nations and the English regions in the entry year or follow-up duration. However, on inspection, this was not the case (online supplementary figures S9 and S10). The sensitivity analysis including patients with early outcomes (THR/TKR within 2 years of index consultation) gave similar levels of discrimination and calibration (online supplementary figure S11 and table S4).”^66^

## TRIPOD-Cluster checklist: discussion

### Item 16a: give an overall interpretation of the main results, including heterogeneity across clusters in model performance, in the context of the objectives and previous studies

Authors should first discuss the main results from their pooled analysis, whether the study objectives (as listed in the introduction) were met, and if not, why not. They should then interpret the study results to place the findings in context of other evidence, including previous studies on different models for the same target population and outcomes and the biological plausibility of new predictors (eg, biomarkers) included in the developed model. Studies using clustered data should also interpret the model’s overall performance in light of the assessed quality of the different data sources. When reflecting on other sources of evidence, the authors should explicitly refer to any prediction models that were developed or validated from the individual component datasets.

Key issues in prediction model studies using clustered data are the feasibility of using the developed or validated model in diverse settings and how it can fit into or alter the target medical practice. Authors should therefore explicitly discuss any observed and quantified heterogeneity in the performance of the developed or validated model across the different data sources, settings, or subgroups.

Authors should discuss whether their model makes accurate enough predictions to recommend its use to readers, care providers, and guideline developers to enhance their decision making. Authors should make clear which contexts the model is useful in, whether they recommend the model before or after updating, and whether the model requires further study or external validation before it can be recommended for use in practice.

#### Example of individual participant data meta-analysis

“We have shown that non-invasive models for prediction of incident type 2 diabetes have acceptable to good discrimination over 10 years, both overall and across countries. After recalibration, most models showed good calibration, which was consistent across countries, although discrimination varied significantly. We showed that the models’ performance is worse in men than in women. Discrimination is better in people younger than 60 years, but risk can be overestimated in this age group. Discrimination is generally lower in participants with a BMI [body mass index] of less than 25 kg/m^2^ than in those with a BMI of at least 25 kg/m^2^. Risk is systematically overestimated in participants with a BMI of less than 25 kg/m^2^. No model significantly outperforms others enough to be uniquely recommended for routine risk stratification . . . A few previous validation studies of incident diabetes models have been done . . . Overall, the previous studies showed that the models had modest to good discrimination and poor calibration. However, intercept adjustment to correct for differences in diabetes incidence between development and validation populations was done in only one study.”^45^

#### Example using electronic health records data

“The COPE [critical care outcome prediction equation] model is a simple mortality prediction tool derived from an administrative database (the VAED [Victorian Admitted Episode Database]) and contains six variables present on admission. The COPE-4 model provides an estimate of the risk of in-hospital death for adult patients admitted to the ICU and performs consistently and satisfactorily in the majority of hospitals across a diverse population . . . The COPE-4 model appeared to underestimate risk in tertiary and metropolitan hospitals and overestimate risk in regional hospitals. In the majority, these deviations were small and much less than those currently observed for the APACHE III-j model. Recalibration and refinement of the two models may address some of these limitations.”^162^

### Item 16b: for validation, discuss the results with reference to the model performance in the development data, and in any previous validations

Authors that validate one or more prediction models using multiple combined data sources should compare their results to previous validations of the same model in other data and to the observed model performance in the original development study. They can also discuss the results of external validation studies of relevant competing models in other datasets to give a more complete picture. All such comparisons are qualitative, because they include indirect comparisons of the performance of a prediction model across different datasets. Nevertheless, they do contribute to the overall picture of the model's generalizability and transportability across different settings, countries, populations, or subpopulations.

#### Example of individual participant data meta-analysis

“We used a pooled individual patient database from five studies as a validation cohort. The ability of the VPM [Vienna prediction model] to distinguish patients’ risk for recurrent VTE [venous thromboembolism] in the validation cohort was at least as good as in the original cohort. The robustness of the VPM in distinguishing between patients at high or low risk of recurrence in the validation cohort is evident, despite differences in patient characteristics in the two cohorts. Hence, patients in the validation cohort were older, had higher D-dimer levels, less often had distal DVT [deep vein thrombosis], and experienced recurrences over a slightly shorter time-to-event.”^183^

#### Example using electronic health records data

“Previous independent validations of models of breast cancer risk include two single-site prospective studies that were included in ProF-SC [Prospective Family Study Cohort], but our study includes data for a further five sites and has much longer follow-up data. A large validation study of 567 prospective invasive cases in a screening cohort of 50 061 women compared IBIS [International Breast Cancer Intervention Study model] (version 6) with BCRAT [Breast Cancer Risk Assessment Tool] and showed improved performance with the pedigree information (ie, with IBIS), but the cohort was only followed up for a median of 3.2 years.”^191^

### Item 16c: discuss the strengths of the study and any limitations (eg, missing or incomplete data, non-representativeness, data harmonisation problems)

A balanced discussion of limitations strengthens, rather than weakens, published research. Even the best conducted prediction model studies are likely to have several limitations. Limitations need to be placed into perspective, and an effort should be made to characterise the possible impact of each problem on the study’s results. Limitations can pertain to any aspect of the study design, conduct, or analysis. Authors should clarify whether each limitation affects model development, validation, or both, and their overall impact on the credibility, applicability, and generalisability of the prediction model. For example, in the context of IPD from multiple studies, predictors might be systematically missing from one or more datasets. Readers need to know about the possible impact this missing data could have on the study findings and how it was handled to properly judge the study. Data harmonisation can induce a loss in precision. The extent to which this occurred should be discussed.

The quality of data from large routinely collected data are often questioned. It is typically entered during a consultation with the care practitioner, and not for the purpose of conducting research. Non-standardised definitions of diagnoses and outcomes, coding of comorbidities (eg, Read codes, ICD-10 codes), lack of recording of potentially important predictors, missing data, and non-representativeness of the sample are all potential limitations of any prediction model study that use routinely collected data.

Authors should also highlight the strengths of their study. For example, large, routinely collected, EHR data provide opportunities to develop and directly validate prediction models that can account for differences across clusters (eg, hospitals or general practices). Heterogeneity in model performance can be explored and sources of heterogeneity investigated.

#### Example of individual participant data meta-analysis

“We combined existing data from several different hospitals. Since the current analysis was not the main purpose of the data collection, the selection of patients, availability of data, and predictor definitions differed across hospitals . . . Overall, heterogeneity due to differences between protocols, level of physician experience, and guideline adherence across hospitals could have influenced our results. Despite these limitations, the models presented had generally good discrimination (via the c-statistic) and calibration. Because we intended to use our model in low prevalence populations, cross validation was performed using only the low prevalence datasets. The cross-validation results in the data from Parma, Rotterdam, and the smaller hospitals combined, were less favourable in terms of calibration-in-the-large, possibly explained by heterogeneity. However, in general, calibration assessed graphically could be considered satisfactory, suggesting that the model is generalisable to other settings. Further external validation of our model in other populations is still needed.”^161^

#### Example using electronic health records data

“A major strength of this study is the size and the representativeness of the cohort, by including a large number of general practices using the EMIS computer system. A limitation of this study is the considerable amounts of missing data for total serum cholesterol to high density lipoprotein ratio both in the derivation and the external validation of QRISK2-2011. Despite the large amounts of missing data, information on all risk factors were available for 400 000 people, and 800 000 people had none or only one missing risk factor. However, we used current recommended approaches with multiple imputation to overcome the biases that occur when omitting people with incomplete data.”^192^

### Item 17: discuss the potential use of the model and implications for future research, with specific view to generalisability and applicability of the model across different settings or (sub)populations

As in similar studies using data from a single cluster, authors of prediction model studies using clustered data should discuss the clinical and research implications of their developed or validated models.^2^ Prediction models can have different purposes and be used at different moments in the targeted individual’s healthcare journey. These elements are important to stress in a discussion. The clinical use of prediction models should only be recommended when evidence shows their likely performance and their potential to improve patient related outcomes or efficiency in healthcare through better decisions in the management of patients.^9^

Prediction model studies using clustered data usually have the unique opportunity to test the studied model’s performance across subsets of the data at hand.^38^ ^46^ Such testing of heterogeneity in the model’s performance can include testing across selected care settings (eg, primary, secondary, emergency, or other types of care), geographical locations, or participant subgroups. Whether and how the model’s performance changes across these tested subsets is important for inferences about the model’s transportability. Authors should therefore discuss heterogeneity in model performance and its effects on transportability.

A model’s effect on decision making, decision making behaviour, and patient outcomes can best be evaluated in comparative (preferably randomised) studies comparing the use of the model and subsequent management against usual care or competing models in a head-to-head fashion.^193^ ^194^ ^195^ Unfortunately, such model impact studies are rare and costly.^196^ ^197^ Explicit decision-analytic modelling studies that model the relation between model performance and the effectiveness of subsequent management decisions (ie, a linked evidence approach) could produce further insight in the uncertainties and whether a model impact study is needed before the model can be recommended for use.^193^ ^198^

#### Example of individual participant data meta-analysis

“Our study brings a simple and user-friendly predictor for sudden death risk, specifically for patients of hypertension . . . The score was built on the point system for an easy assessment of a hypertensive individual's risk of sudden death in 5 years . . . Our HYSUD [sudden death risk for hypertension] score was built from a database collected in the period of 1970 – 1990, similarly to classical scores such as Framingham or Systemic Coronary Risk Estimation and hence, should be calibrated before application for nowadays patients, to limit possible bias coming from change in covariable hazards ratio over time or other reasons.”^199^

#### Example using electronic health records data

“Our newly developed risk algorithms could have important applications in clinical practice by helping direct annual monitoring, intensive non-surgical care and timely assessment and discussion of the need for surgical referral to those most at risk of progression. The algorithms can specifically identify the individuals who, in the context of current healthcare policies and resources, are at higher risk of future joint replacement, and therefore can be targeted for individual care ranging from earlier surgery to non-invasive care that might postpone the need for surgery. The hypothetical higher risk individual . . . might, for instance, be targeted for a programme of more intensive multimodal therapy including graded supervised exercise and supported weight loss. The algorithm also uses future joint replacement as a proxy for future progression of osteoarthritis, and therefore potentially attempting to identify individuals more broadly who can be targeted for more intensive monitoring and interventions that might prevent such future progressions and severity regardless of whether they would actually have had a joint replacement.”^66^

## TRIPOD-Cluster checklist: other information

### Item 18: provide information about the availability of supplementary resources (eg, study protocol, analysis code, datasets)

All research on humans should ideally be protocol driven, and prediction model studies involving clustered data are no exception.^200^ In fact, protocols might be even more beneficial for these studies, given the complexity and many choices that are typically present. Researchers are not currently required to register observational studies, with recent support for 201 202 203 204 and opposition against this idea.^205^ ^206^ ^207^ Many clinical trial registries, including ClinicalTrials.gov (https://www.clinicaltrials.gov/), explicitly state that observational studies can be registered.^208^ Researchers using multiple data sources can also register their studies on PROSPERO (https://www.crd.york.ac.uk/prospero/), an international prospective register of systematic reviews.

Potential users of a prediction model need the full details of the model to be published (item 12b). We therefore recommend researchers to provide all analysis code, including steps taken for data extraction, data processing, model fitting, and model evaluation. Authors are recommended to also provide links for accessing any web calculators or standalone applications that have been developed.^164^ The research community is increasingly supportive of making datasets and computer code publicly available for reproducing analyses.^209^ ^210^ ^211^ ^212^ Further useful information can be found in the original TRIPOD E&E and on www.tripod-statement.org.^2^

#### Example of individual participant data meta-analysis

“A protocol for this systematic review was submitted to the National Institute for Health Research, outlining the methods which follow, published in BioMed Central Systematic Reviews journal and was registered on PROSPERO (CRD42013003494).”^98^

#### Example using electronic health records data

“The study is being performed by the ISARIC Coronavirus Clinical Characterisation Consortium (ISARIC-4C) in 260 hospitals across England, Scotland, and Wales (National Institute for Health Research Clinical Research Network Central Portfolio Management System ID 14152). The protocol and further study details are available online.”^213^

### Item 19: give the source of funding and the role of the funders for the present study

Authors should disclose all sources of funding received for conducting the study and state what role each funder had in the study’s design, conduct, analysis, and reporting. If the funders had no involvement, the authors should say so. Similarly, if the study received no funding, the authors should clearly say so.

#### Example of individual participant data meta-analysis

“This study was funded by Federal Ministry of Education and Research, Germany (BMBF–grant no. FKZ 01GK0920). The funding source had no involvement in the study.”^65^

#### Example using electronic health records data

“This study is supported by the UK Biotechnology and Biological Sciences Research Council grant number BBSRC BB/M009513/1 to SH. The funder plays no role in the design of this study, data collection, data analysis, interpretation of data, writing of the report or in the decision to submit the protocol for publication.”^99^

## Web extra

Extra material supplied by authors

Web appendix: Supplementary table - TRIPOD-Cluster checklist

## Notes

Contributors: TPAD is guarantor. TPAD, GSC, RDR, Doug Altman, JBR, and KGMM conceived the TRIPOD-Cluster statement and developed an initial checklist. TPAD organised two Delphi rounds to obtain feedback on individual items of the TRIPOD-Cluster checklist. TPAD, KGMM, RDR, and GSC prepared the first draft of this manuscript. All authors reviewed subsequent drafts and assisted in revising the manuscript.The corresponding author attests that all listed authors meet authorship criteria and that no others meeting the criteria have been omitted.
