# Supplementary material for: Transparent reporting of multivariable prediction models developed or validated using clustered data (TRIPOD-Cluster): explanation and elaboration
Source: BMJ. 2023 Feb 7;380:e071058. doi: 10.1136/bmj-2022-071058 (PMC9903176; doi:10.1136/bmj-2022-071058)
Supplement: Supplementary file 1 — Web appendix: Supplementary table - TRIPOD-Cluster checklist [file debt071058.ww.pdf]

Supplementary table: Checklist of items to include when reporting a study developing or validating a multivariable prediction model using clustered data (TRIPOD-Cluster)

Please cite: Debray TPA, Collins GS, Riley RD, et al. Transparent reporting of multivariable prediction models developed or validated using clustered data (TRIPOD-Cluster): explanation and elaboration. *BMJ* 2023;380:e071058. doi:10.1136/bmj-2022-071058.

Section / Topic	#	Description	Page #
**Title and Abstract**			
Title	1	Identify the study as developing and/or validating a multivariable prediction model, the target population, and the outcome to be predicted.	
Abstract	2	Provide a summary of research objectives, setting, participants, data source, sample size, predictors, outcome, statistical analysis, results, and conclusions.*	
**Introduction**			
Background and objectives	3a	Explain the medical context (including whether diagnostic or prognostic) and rationale for developing or validating the prediction model, including references to existing models, and the advantages of the study design. *	
	3b	Specify the objectives, including whether the study describes the development or validation of the model. *	
**Methods**			
Participants and data	4a	Describe eligibility criteria for participants and datasets. *	
	4b	Describe the origin of the data, and how the data were identified, requested and collected.	
Sample size	5	Explain how the sample size was arrived at. *	
Outcomes and predictors	6a	Define the outcome that is predicted by the model, including how and when assessed. *	
	6b	Define all predictors used in developing or validating the model, including how and when measured. *	
Data preparation	7a	Describe how the data were prepared for analysis, including any cleaning, harmonisation, linkage, and quality checks.	
	7b	Describe the method for assessing risk of bias and applicability in the individual clusters (e.g., using PROBAST).	

	7c	For validation, identify any differences in definition and measurement from the development data (e.g., setting, eligibility criteria, outcome, predictors). *	
	7d	Describe how missing data were handled. *	
Data analysis	8a	Describe how predictors were handled in the analyses.	
	8b	Specify the type of model, all model-building procedures (e.g. any predictor selection and penalization), and method for validation. *	
	8c	Describe how any heterogeneity across clusters (e.g., studies or settings) in model parameter values was handled.	
	8d	For validation, describe how the predictions were calculated.	
	8e	Specify all measures used to assess model performance (e.g. calibration, discrimination, and decision curve analysis) and, if relevant, to compare multiple models.	
	8f	Describe how any heterogeneity across clusters (e.g., studies or settings) in model performance was handled and quantified.	
	8g	Describe any model updating (e.g., recalibration) arising from the validation, either overall or for particular populations or settings. *	
Sensitivity analysis	9	Describe any planned subgroup or sensitivity analysis, e.g. assessing performance according to sources of bias, participant characteristics, setting.	
**Results**			
Participants and datasets	10a	Describe the number of clusters and participants from data identified through to data analysed. A flow chart may be helpful.*	
	10b	Report the characteristics overall and where applicable for each data source or setting, including the key dates, predictors, treatments received, sample size, number of outcome events, follow-up time, and amount of missing data.*	
	10c	For validation, show a comparison with the development data of the distribution of important variables (demographics, predictors, and outcome).	
Risk of bias	11	Report the results of the risk of bias assessment in the individual clusters.	
Model development and specification	12a	Report the results of any across-cluster heterogeneity assessments that led to subsequent actions during the model’s development (e.g., inclusion or exclusion of particular predictors or clusters).	
	12b	Present the final prediction model (i.e., all regression coefficients, and model intercept or baseline estimate of the outcome at a given	

		time point) and explain how to use it for predictions in new individuals.*	
Model performance	13a	Report performance measures (with uncertainty intervals) for the prediction model, overall and for each cluster.	
	13b	Report results of any heterogeneity across clusters in model performance	
Model updating	14	Report the results from any model updating (including the updated model equation and subsequent performance), overall and for each cluster.*	
Sensitivity analysis	15	Report results from any subgroup or sensitivity analysis.	
**Discussion**			
Interpretation	16a	Give an overall interpretation of the main results, including heterogeneity across clusters in model performance, in the context of the objectives and previous studies.*	
	16b	For validation, discuss the results with reference to the model performance in the development data, and in any previous validations.	
	16c	Discuss the strengths of the study and any limitations (e.g. missing or incomplete data, non-representativeness, data harmonisation problems).*	
Implications	17	Discuss the potential use of the model and implications for future research, with specific view to generalizability and applicability of the model across different settings or (sub)populations.*	
**Other information**			
Supplementary Information	18	Provide information about the availability of supplementary resources (e.g., study protocol, analysis code, data sets). *	
Funding	19	Give the source of funding and the role of the funders for the present study.	
